# Diversification, Biogeographic Pattern, and Demographic History of Taiwanese *Scutellaria* Species Inferred from Nuclear and Chloroplast DNA

**DOI:** 10.1371/journal.pone.0050844

**Published:** 2012-11-30

**Authors:** Yu-Chung Chiang, Bing-Hong Huang, Pei-Chun Liao

**Affiliations:** 1 Department of Biological Sciences, National Sun Yat-sen University, Kaohsiung, Taiwan; 2 Department of Biological Science and Technology, National Pingtung University of Science and Technology, Pingtung, Taiwan; George Washington University, United States of America

## Abstract

The ragged topography created by orogenesis generates diversified habitats for plants in Taiwan. In addition to colonization from nearby mainland China, high species diversity and endemism of plants is also present in Taiwan. Five of the seven *Scutellaria* species (Lamiaceae) in Taiwan, for example, are endemic to the island. Hypotheses of multiple sources or *in situ* radiation have arisen to explain the high endemism of Taiwanese species. In this study, phylogenetic analyses using both nuclear and chloroplast markers revealed the multiple sources of Taiwanese *Scutellaria* species and confirmed the rapid and recent speciation of endemic species, especially those of the “indica group” composed of *S. indica*, *S. austrotaiwanensis*, *S. tashiroi*, and *S. playfairii*. The common ancestors of the indica group colonized first in northern Taiwan and dispersed regionally southward and eastward. Climate changes during glacial/interglacial cycles led to gradual colonization and variance events in the ancestors of these species, resulting in the present distribution and genetic differentiation of extant populations. Population decline was also detected in *S. indica*, which might reflect a bottleneck effect from the glacials. In contrast, the recently speciated endemic members of the indica group have not had enough time to accumulate much genetic variation and are thus genetically insensitive to demographic fluctuations, but the extant lineages were spatially expanded in the coalescent process. This study integrated phylogenetic and population genetic analyses to illustrate the evolutionary history of Taiwanese *Scutellaria* of high endemism and may be indicative of the diversification mechanism of plants on continental islands.

## Introduction

Radiation and colonization are two major mechanisms for the development of high diversity in continental islands [1], [2]. The term radiation denotes a phenomenon of rapid speciation in a specific locality from a single origin, also defined as *in situ* diversification, which fits the propagule pool model in displaying single-source colonization [3]. By contrast, colonization is the phenomenon of species originating from multiple sources (or multiple origination), which can be illustrated by the migrant pool model [3]–e.g., the plant diversity of Taiwan and Ryukyu Archipelago [4]. However, whether through single or multiple originations, the appearance of endemic species on a continental island must involve reproductive isolation from the closely related species of continents.

Taiwan is a continental island situated off of Southeast Asia. The emergence of the shallow shelf of the Taiwan Strait during the Pleistocene glacial cycles connected Taiwan Island and mainland China, whereas the submergence of the shallow continental shelf during the interglacial periods separated them. The repeated topographic changes during the Pleistocene glacials resulted in several opportunities for colonization and isolation of organisms between China and Taiwan [4]. Such biogeographic events could cause that 52% of the native plant species of Taiwan have affinities to the flora of mainland China and high endemism of flora in Taiwan (*c.* 26.1% of natives) [5]. Therefore, high endemism in the flora of Taiwan can be explained by hypotheses of (1) continent-island colonization, (2) *in situ* radiation, or (3) synergy of colonization and radiation. The colonization hypothesis focuses on the phenomenon of multiple colonization events via the land bridge at the Taiwan Strait during the glacials, whereas the radiation hypothesis focuses on *in situ* speciation after colonization in Taiwan. The radiation hypothesis is usually related to local adaptation (i.e. adaptive radiation [6], [7]) or drift effect by geographic isolation and demographic change (i.e. non-adaptive radiation [8]). Although Taiwan is subtropical and was free of ice during the Pleistocene glacials, the unpleasant cold and dry conditions affected the demography of several Taiwanese organisms [9]–[15]. The rapid process of genetic drift of small-sized populations in glacial refugia can enlarge the gap between phylogenetically related “pre-species” [16], [17]. To explore the mechanism of formation of the endemics, we chose species of the genus *Scutellaria* as models in which to test the continent-island colonization hypothesis and the radiation (adaptive or non-adaptive) hypothesis.

*Scutellaria*, a genus commonly known as skullcaps, contains approximately 400 species around the world. *Scutellaria* is sister to *Tinnea* and together they form the well-supported group Scutellarioideae [18]. Several members of this genus–e.g., *S. baicalensis*, *S. indica* and *S. lateriflora–*are widely used in traditional medicine [19]–[23]. The specific floral types of both cleistogamous and chasmogamous flowers [24], [25] and restricted seed dispersal capabilities of bursting capsules [26]–[28] may have caused structured populations, especially in cases of widely distributed species, resulting in the high diversity of this genus. For example, predominant selfing, even in chasmogamous flowers, increases genetic differentiation between populations of *S. indica* [24]. Furthermore, the loss of pollinators for historical outcrossing populations has often been reported in *Scutellaria montana*, which do not produce cleistogamous flowers [28]. Similar patterns of restricted outcrossing are also observed in Taiwanese *Scutellaria* species (personal observation).

Five of seven *Scutellaria* species, *S. taipenensis, S. playfairii*, *S. tashiroi*, *S. austrotaiwanensis*, and *S. taiwanensis*, are endemic to Taiwan, while the other two species, *S. barbata* and *S. indica*, are widespread in Asia and are treated in the same section, Sect. *Scutellaria* [29]. Most of them are distributed between central and southern Taiwan [30]–[32] except the northerly distributed *S. taipeiensis* Huang, Hsiao, *et* Wu [33]. The Taiwanese species are morphologically similar and have identical chromosome numbers (2n = 26) [30]–[33], which fit the chromosome range of Sect. *Scutellaria* (2n = 24∼34) [29]. Mainland China, which contains 124 taxa (98 species and 26 varieties) [34], is probably the source of the Taiwanese species. Until recently, three endemic species, *S. tashiroi*, *S. playfairii*, and *S. austrotaiwanensis*, were considered different varieties under a species level [35],[31]. The same corolla type is also shared among *S. indica*, *S. playfairii*, and *S. austrotaiwanensis* [30], [31]. Some older specimens or records identified as *S. indica* in South Taiwan have been proposed to be misidentifications of *S. taiwanensis* [30]. These patterns imply a close phylogenetic relationship among *S. tashiroi*, *S. playfairii*, *S. austrotaiwanensis*, *S. indica*, and *S. taiwanensis*. These five species have more or less overlapping distributions but may differ in microhabitat preference. For example, *S. playfairii* is distributed in southern and eastern Taiwan, and *S. tashiroi* is distributed in eastern Taiwan and Lanyu Island. Both species can be found sympatrically in eastern Taiwan, but *S. tashiroi* prefers open rocky slopes, whereas *S. playfairii* prefers more or less shady slopes. *Scutellaria austrotaiwanensis* is mainly distributed in the Hengchun Peninsula, but some small patchy populations are also found in other region in southern Taiwan. *Scutellaria taiwanensis* grows in a habitat similar to that of *S. austrotaiwanensis* and *S. playfairii* in southern Taiwan but prefers more moist and shaded underforest habitats at slightly higher elevations. *Scutellaria indica*, which is not endemic to Taiwan, is distributed in northeastern and western Taiwan and is geographically differentiated from other species [30], [31].

To evaluate the hypotheses of diversification of Taiwanese *Scutellaria*, we designed both species-level and population-level studies to explore the evolutionary history of the genus. Phylogenetic analyses for Taiwanese *Scutellaria* were performed with several species collected from Japan, Asian mainland, Europe, North America, and South America to confirm the continent-island colonization hypothesis. Demographic change and biogeographic events in endemic *Scutellaria* species were further examined to test the radiation hypothesis. Based on these phylogenetic and population genetic analyses, we tried to determine the evolutionary history of Taiwanese *Scutellaria* species to elucidate mechanisms of diversification in continental island herbs.

## Materials and Methods

### Taxon Sampling and Study Populations

**Figure 1:**
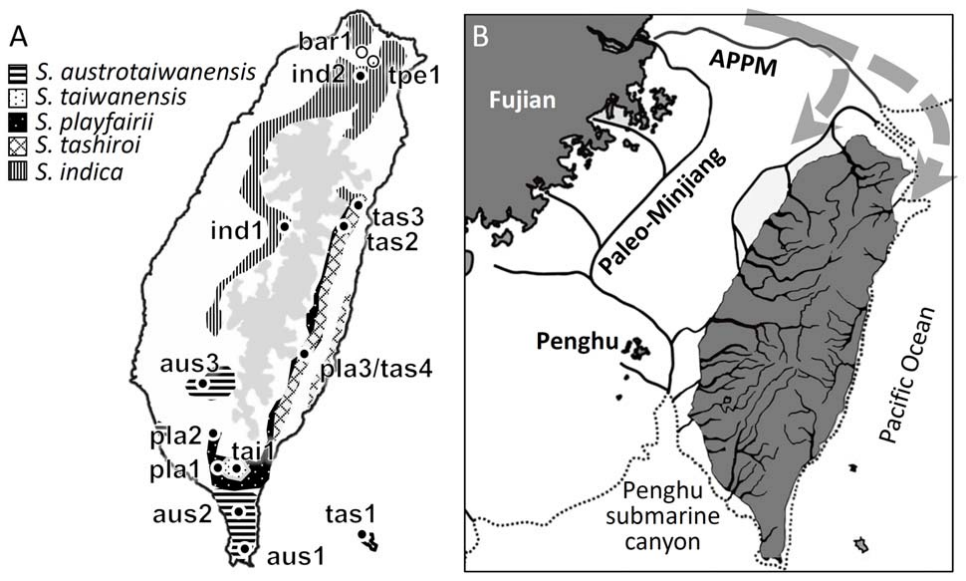
Species distribution and the paleo-drainage system in Taiwan. (A) Distribution areas and sampling populations (solid dots) of five Taiwanese *Scutellaria* species used for demographic analyses. The sampling sites Yonghe (bar1) and Maokong (tpe1) for *S. barbata* and *S. taipeiensis*, respectively, are also indicated on this map (hollow dots). The gray areas are mountain ranges with altitudes of >2000 m. Aus, tai, pla, tas, ind, bar, and tpe indicate the sampling sites of *S. austrotaiwanensis*, *S. taiwanensis*, *S*. *playfairii*, *S. tashiroi*, *S. indica*, *S. barbata*, and *S. taipeiensis*, respectively. Detailed information of sampling sites is listed in Table 1. (B) The proposed paleo-drainage system around the Taiwan Strait during glacial periods of the Late Pleistocene [79]. The dotted arrows are possible colonization routes of ancestors of the indica group; the dotted line indicates the Late Pleistocene shoreline. APPM, the alternate path of the Paleo-Minjiang River.

To resolve the hypotheses of multiple sources or single origin of Taiwanese *Scutellaria* species, we sampled all seven Taiwanese *Scutellaria* species and an additional 10 species in the field or from the Seed Bank, including *S. amoena*, *S. amabilis*, *S. sessilifolia*, *S. galericulata*, *S. lateriflora*, *S. incana*, *S. alpine*, *S. baicalensis*, *S. salviifolia*, *S. diffusa*, *S. altissima*, and *S. zhongdianensis* (Table S1). *Tinnea rhodesiana* was used as the outgroup. Population sampling was also performed for *S. indica*, *S. tashiroi*, *S. austrotaiwanensis*, and *S. playfairii* (namely, the indica group; see Results) and *S. taiwanensis* to reconstruct their biogeographic patterns (Table 1 and Figure 1A). In total, 269 individuals from 13 populations were collected. This study was conducted in accordance with the laws of Taiwan. The locations of field studies are not privately-owned or protected areas, and are not involved with endangered or protected species. No permits were required for this study. Leaves obtained from each individual were dried with silica gel immediately for preservation. The dried materials were ground to powder using liquid nitrogen and extracted with a cetyl trimethylammonium bromide method [36]. The extracted DNA was then dissolved in 1X TE buffer and stored at −20°C.

**Table 1:** Sampling areas and haplotype composition of *Scutellaria* species in the population study.

Species/population	Population code	Sample size	Longitude	Latitude	Haplotype size	Haplotype compositionof each locus				
						*CHS*	*CAD*	*mat*K	*ndh-rpl32*	*rpl32-trnL*
***S. indica*** †										
Awanda	ind1	9	121° 10′ 31′′E	23° 57′ 12′′N	18	CHS-hap3	CAD-hap5	matK-hap2	ndh/rpl-hap2	rpl/trn-hap2
Wulai	ind2	12	121° 34′ 27′′E	24° 53 ′07′′N	24	CHS-hap4	CAD-hap6	matK-hap2	ndh/rpl-hap3	rpl/trn-hap3
***S. austrotaiwanensis*** * †										
Hengchun peninsula	aus1	19	120° 52′ 24′′E	22° 00′ 34′′N	38	CHS-hap2	CAD-hap4	matK-hap1	ndh/rpl-hap1	rpl/trn-hap1
Lilungshan	aus2	33	120° 43′ 11′′E	22° 10′ 35′′N	42	CHS-hap1	CAD-hap1	matK-hap1	ndh/rpl-hap1	rpl/trn-hap1
					24	CHS-hap1	CAD-hap2	matK-hap1	ndh/rpl-hap1	rpl/trn-hap1
Nanhua	aus3	14	120° 21′ 40′′E	23° 01′ 07′′N	28	CHS-hap1	CAD-hap3	matK-hap1	ndh/rpl-hap1	rpl/trn-hap1
***S. playfairii*** * †										
Dahan trail	pla1	5	120° 45′ 17′′E	22° 24′ 46′′N	10	CHS-hap5	CAD-hap7	matK-hap1	ndh/rpl-hap4	rpl/trn-hap6
Wutai	pla2	43	120° 43′ 05′′E	22° 44′ 23′′N	38	CHS-hap5	CAD-hap7	matK-hap1	ndh/rpl-hap4	rpl/trn-hap4
					14	CHS-hap5	CAD-hap7	matK-hap1	ndh/rpl-hap4	rpl/trn-hap5
					22	CHS-hap5	CAD-hap7	matK-hap1	ndh/rpl-hap5	rpl/trn-hap4
					4	CHS-hap5	CAD-hap7	matK-hap1	ndh/rpl-hap5	rpl/trn-hap5
					4	CHS-hap5	CAD-hap8	matK-hap1	ndh/rpl-hap4	rpl/trn-hap4
					2	CHS-hap5	CAD-hap8	matK-hap1	ndh/rpl-hap4	rpl/trn-hap5
					2	CHS-hap5	CAD-hap8	matK-hap1	ndh/rpl-hap5	rpl/trn-hap4
Wulu	pla3	35	121° 02′ 30′′E	23° 10′ 26′′N	46	CHS-hap6	CAD-hap9	matK-hap1	ndh/rpl-hap4	rpl/trn-hap5
					16	CHS-hap6	CAD-hap9	matK-hap1	ndh/rpl-hap4	rpl/trn-hap6
					8	CHS-hap6	CAD-hap9	matK-hap1	ndh/rpl-hap6	rpl/trn-hap5
***S. tashiroi*** * †										
Lanyu Island	tas1	15	121° 30′ 40′′E	22° 04′ 32′′N	30	CHS-hap10	CAD-hap12	matK-hap1	ndh/rpl-hap10	rpl/trn-hap1
Mugumuyu	tas2	16	121° 25′ 35′′E	24° 00′ 10′′N	32	CHS-hap10	CAD-hap13	matK-hap1	ndh/rpl-hap11	rpl/trn-hap8
Taroko	tas3	15	121° 30′ 51′′E	24° 10′ 42′′N	10	CHS-hap10	CAD-hap13	matK-hap1	ndh/rpl-hap11	rpl/trn-hap8
					20	CHS-hap11	CAD-hap13	matK-hap1	ndh/rpl-hap11	rpl/trn-hap8
Wulu	tas4	22	121° 02′ 30′′E	23° 10′ 26′′N	12	CHS-hap8	CAD-hap11	matK-hap1	ndh/rpl-hap9	rpl/trn-hap1
					10	CHS-hap9	CAD-hap11	matK-hap1	ndh/rpl-hap1	rpl/trn-hap1
					12	CHS-hap9	CAD-hap11	matK-hap1	ndh/rpl-hap4	rpl/trn-hap1
					6	CHS-hap9	CAD-hap11	matK-hap1	ndh/rpl-hap9	rpl/trn-hap1
					2	CHS-hap12	CAD-hap11	matK-hap1	ndh/rpl-hap4	rpl/trn-hap1
					2	CHS-hap13	CAD-hap11	matK-hap1	ndh/rpl-hap4	rpl/trn-hap1
***S. taiwanensis*** *										
Jin-Shui Camp	tai1	31	120° 43′ 42′′E	22° 24′ 52′′N	20	CHS-hap7	CAD-hap10	matK-hap3	ndh/rpl-hap7	rpl/trn-hap7
					42	CHS-hap7	CAD-hap10	matK-hap3	ndh/rpl-hap8	rpl/trn-hap7

* endemic to Taiwan;

† members of the indica group.

### Molecular Techniques

Polymerase chain reaction (PCR) was performed with 10–100 ng template DNA, 0.5–1 U Taq (Bernardo Scientific Corp., Taipei), 100 µM deoxyribonucleotide triphosphate, 0.2 µM each primer, and 0.1 µg/µL bovine serum albumin in a MultiGene thermal cycler (Labnet International, Inc.). The PCR program was set to 94°C for 3 min for enzyme activation, followed by 35 cycles of 94°C for 40 s, melting temperature for 40 s, and 72°C for 90 s, with a 5-min final extension at 72°C. PCR amplifications of five primer sets including three chloroplast regions (*mat*K, *ndh*F-*rpl*32, and *rpl*32-*trn*L) and two low-copy nuclear regions (*CHS* and *CAD*) were performed. Optimal annealing temperatures were set at 47°C for chloroplast regions, 53°C for CHS, and 49°C for CAD regions. Within-population variation of all PCR products was screened with single-strand conformation polymorphism. Each PCR product was denatured for 10 min at 95°C and quickly moved into a −20°C cool box. Denatured products were separated by pre-cooling 10% polyacrylamide gel (acrylamide:bisacrylamide = 45∶1). PCR products with different fragment patterns were then sequenced directly in both directions using an ABI BigDye 3.1 Terminator Cycle Sequencing Kit (Applied Biosystems, Foster City, CA, USA). All sequence polymorphisms were visually rechecked from chromatograms with an ABI PRISM®3730XL DNA Sequencer (Perkin-Elmer, Foster City, CA, USA). PCR products were cloned with a yT&A cloning kit (Yeastern Biotech, Taipei, Taiwan) when they contained ambiguous nucleotides, and three to five clones were sequenced with the M13F and M13R primers to generate consensus sequences. The two sequences of a heterozygote were separated by comparing the sequences of the PCR product and the cloned sequence. Chromatograms were inspected by SeqMan implemented in DNASTAR ver. 7.0 (Lasergene, Germany). Gene confirmation and exon-intron junctions of each sequence were queried in the Nucleotide collection database at the National Center for Biotechnology Information website using the Nucleotide Basic Local Alignment Search Tool program and NetPlantGene server at the Center for Biological Sequence Analysis website (www.cbs.dtu.dk/services/NetPGene/). All sequences were deposited in the NCBI nucleotide sequence database under the following accession numbers: JX981343∼JX981446 and JX985445∼JX985457.

### Phylogenetic Reconstruction

In addition to collecting sequences, we also downloaded the *mat*K sequences of *S. minor* (HM850804.1), *S. scordifolia* (HQ839713.1), *S. hirta* (HQ911383.1), *S. sieberi* (HQ911384.1), *S. viscidula* (HQ676587.1), *S. rehderiana* (HQ676588.1), *T. gracilis* (HQ911386.1, the outgroup used for *mat*K only), and the *CHS* sequence of *S. viscidula* (EU386767.1) from GenBank for phylogenetic analyses. Sequence alignments were performed with Clustal X [37] and manually edited using BioEdit ver. 7.0.9.0 [38]. Phylogenetic relationships were reconstructed using individual and combined sequences of five loci with the neighbor joining and Bayesian approaches implemented in Molecular Evolutionary Genetics Analysis v. 5.05 [39] and MrBayes ver. 3.1.2 [40], respectively. In the neighbor-joining analysis, the maximum composite likelihood model for substitutions and pairwise deletions for the treatment of gaps were set with 1000 bootstrap replications to measure the supporting values for grouping. In the Bayesian analysis, substitution models were set according to the evaluation of Akaike information criterion (AIC) and Bayesian information criterion (BIC) scores (Table S2), and two parallel runs in Markov chain Monte Carlo (MCMC) searches were performed for 10 million generations with sampling every 1000 generations for a total of 10,000 trees in each run. The first 10% of the generations were discarded (burn in). Bayesian posterior probabilities were estimated as the proportion of trees containing each node over the trees sampled during runs.

The species tree was also reconstructed using five loci with the assistance of BEAST ver. 1.6.1 [41]. Substitution models of each of loci were set according to the evaluation of AIC and BIC scores (see Table S2). Because we lacked fossil records for dating, we adopted the strict molecular clock in BEAST ver. 1.6.1 using a substitution rate of 2×10^−9^ per site per year [42] for chloroplast genome DNA (cpDNA) loci and 1.5×10^−8^ per site per year [43] for *CHS* and *CAD* with the Yule tree prior [41]. Three independent pre-runs of 10 million generations of the length of the MCMC searches were performed to obtain better parameter priors for the next five independent 10 million generations of the MCMC process. Genealogies were sampled every 1000 generations, with the first 10% discarded as burn in. All the statistics of the output values were summarized using TRACER ver. 1.5 [44] and both log and tree files of the last five runs were combined using LogCombiner ver. 1.6.1 [41]. TreeAnnotator ver. 1.6.1 [41] and FigTree ver. 1.3.1 [45] were used for summarizing and displaying the sampled trees, respectively.

### Topological Tests for Origination Hypotheses

**Figure 2:**
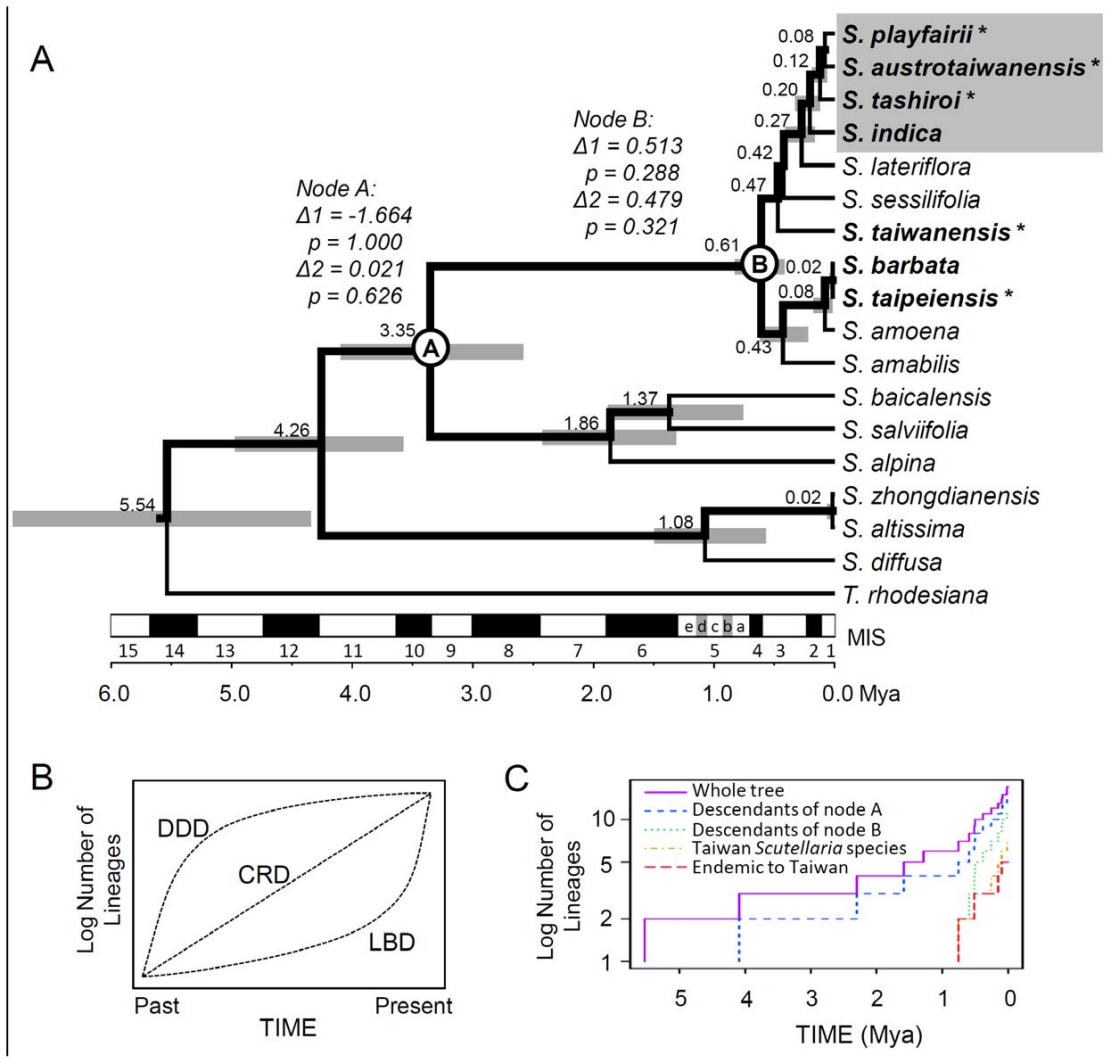
Phylogenetic relationships and the lineage through time (LTT) plots of *Scutellaria* samples in Taiwan. (A) Phylogenetic tree reconstructed using *CHS*, *CAD*, *mat*K, *ndhF-rpl32*, and *rpl*32-*trn*L under the Yule’s pure-birth speciation model. Bold lines indicate lineage grouping with a posterior probability of >90%; the node labels are the splitting time (unit: Mya); the node bar is the 95% highest posterior density interval of the splitting time; species displayed in bold are those distributed in Taiwan; and the stars indicate species endemic to Taiwan. Species inside the gray box are the indica group. The nodes have inferred diversification rate shifts labeled by nodes A and B. The results of the testing of diversification rate shifts of descendants of nodes A and B inferred by Δ-statistics are indicated with italics. Marine Isotope Stages (MIS) are indicated on the time scales. (B) Idealized log LTT plot showing the expected pattern under the hypotheses of density dependent diversification (DDD), constant rate of diversification (CRD), and late burst of diversification (LBD). (C) The lineage through time (LTT) plot of all *Scutellaria* samples used in the study. The graph reveals a delayed increase in the lineage accumulation rate in Taiwan species compared to that in descendants of node B, indicating a short history of species colonizing Taiwan.

**Figure 3:**
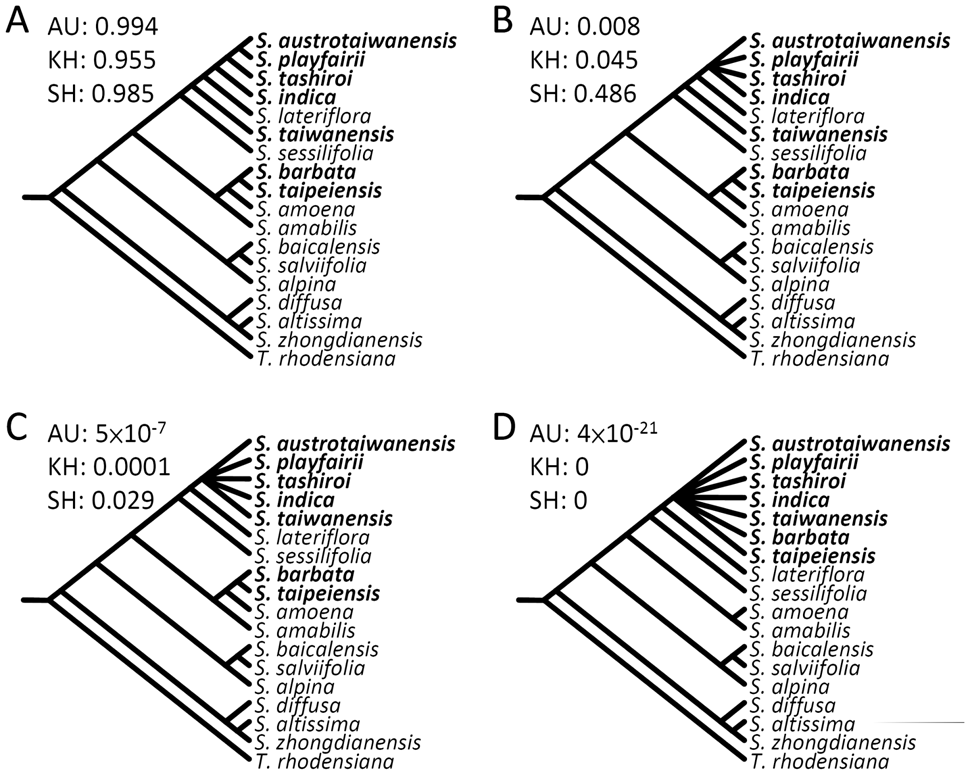
Topological tests of the origin hypotheses of the Taiwan *Scutellaria* species. (A) the Yule speciation tree; (B) hypothesis of single origin of *S. austrotaiwanensis*, *S. playfairii*, *S. tashiroi*, and *S. indica*; (C) *S. taiwanensis* was hypothesized to be singly originated with *S. austrotaiwanensis*, *S. playfairii*, *S. tashiroi*, and *S. indica*; (D) hypothesis of single origin of all Taiwan *Scutellaria* species. Species native to Taiwan are marked in bold. *P* values of the approximately unbiased (AU), Kishino-Hasegawa (KH), and Shimodaira-Hasegawa (SH) tests are indicated.

Given that the phylogenetic analyses indicated that the Taiwanese *Scutellaria* species are not monophyletic (see Results; Figure 2A), topological tests were performed to examine the hypothesis that the Taiwanese species are descendants of a single common ancestor colonizing Taiwan (Figure 3). The approximately unbiased (AU) test [46], the Kishino-Hasegawa (KH) test [47], and the Shimodaira-Hasegawa (SH) test [48], which are used to compare tree topologies with a null hypothesis, were performed using CONSEL [49], [50].

### Population Genetic Diversity of Five Scutellaria Species in Taiwan

To understand better the demographic history of the *Scutellaria* species in Taiwan after colonization, we used the gene markers for phylogenetic analysis and performed population sampling and single-strand conformation polymorphism experiments to obtain the gene frequencies of populations. After sequence alignment, indels were treated as the fifth character when calculating the indices of genetic diversity. The haplotype diversity (*h*), nucleotide diversity estimated by pairwise differences (*π*) and the *θ*_W_ estimated by segregating sites were calculated using the DnaSP ver. 5.10.01 [51]. Both Tajima’s [52] *D* and Fu’s [53] *F*s statistics, which evaluate the degrees of rare alleles and singletons, respectively, were estimated with 1000 coalescent simulations for both individual species and individual populations to assess demographic changes.

### Biogeographic Inference from Statistical Dispersal-vicariance Analysis (S-DIVA)

To understand the biogeographic events of Taiwanese *Scutellaria* species and clarify their origin, we performed S-DIVA [54] to reconstruct the historical geographic ranges of species of the indica group using the RASP program [54]. S-DIVA reconstructs ancestral states with given phylogenetic tree based on Bayesian statistic dispersal-vicariance analysis. It optimized uncertainty of biogeographic events for each node. Because each population of the indica group has a haplotype that differs from the others, we used the “population” as the operational taxonomic units (OTUs) to reconstruct the prior tree of S-DIVA with BEAST using the same approaches as those for the *Scutellaria* species tree. Geographic distributions of the indica group in Taiwan were categorized into the north (population Wulai [ind2] of *S. indica*), the east (Mugumuyu [tas2] and Taroko [tas3] of *S. tashiroi*), the southeast (Wulu of *S. tashiroi* [tas4] and *S. playfairii* [pla3]), the west (Awanda [ind1] of *S. indica*), the south (Dahan Trail [pla1] and Wutai [pla2] of *S. playfairii* and Hengchun peninsula [aus1] and Lilungshan [aus2] of *S. austrotaiwanensis*), the southwest (Nanhua [aus3] of *S. austrotaiwanensis*), and Lanyu Island [tas1] of *S. tashiroi*. Population Jin-Shui Camp [tai1] of *S. taiwanensis* in the south was used as the outgroup to root the indica group. Only the neighboring ancestral areas were included to prevent the unreasonable inference of cross-Central-Mountain-Range dispersal. Information about geographic distributions was referred to the GBIF data portal (data.gbif.org) and specimen records. In total, 10,000 trees from the BEAST MCMC outputs were used, and the annotated tree devised using BEAST was set as the final condensed tree. The number of maximum areas was maintained at three.

### Diversification Rate Shift

The prior tree used for S-DIVA was also used to infer the rate of species occurrence in Taiwan. Both topological and temporal strategies were used to infer diversification rate variation through time. First, the asymmetric rate shift of nodes of the species tree was estimated using single-tree analysis. The taxon-size-insensitive (TSI) and equal-rate Markov (ERM) random branching models were selected. The ERM branching process is a continuous-time method that uses discrete state and pure-birth processes to estimate diversification rate variation in supertrees. Colless’s tree imbalance index and the nodal probability product (*M*_Π_ and its modified version, *M*_Π_*) and sum (*M*_Σ_ and *M*_Σ_*) were used to display the diversification rate variation of the whole tree. Homogeneous evolutionary rates of descendant clades from the common ancestor (the node) were tested with delta-shift statistics (Δ1 and Δ2) at all nodes [55]. One million random resolutions with 1000 TSI-ERM resolved trees under one million ERM simulations were performed to estimate the probabilities of diversification rate shift for each node.

Second, a temporal analysis that accumulated speciation events (lineages) through time was performed to infer the time of species occurrence in Taiwan with lineage through time (LTT) analysis. The LTT analysis was performed in R using the APE package [56]. Chronograms of the species tree reconstructed using BEAST were used as input trees. Sets of total samples of *Scutellaria*, descendants of nodes A and B, and species distributed in Taiwan and endemic to Taiwan were plotted separately for comparison.

### Population Structure

To examine the hierarchical genetic structure of Taiwanese *Scutellaria*, analysis of molecular variance (AMOVA) was performed using Arlequin v. 3.5.1.3 [57]. According to the grouping pattern determined in the phylogenetic analysis, AMOVA was performed using population data from five (the indica group and *S. taiwanensis*) and four (the indica group) species separately. One thousand permutations were executed to evaluate whether the variation distribution of populations/species departed from random variation.

### Extended Bayesian Skyline Analysis

To infer changes in historical demography of five Taiwanese *Scutellaria* species, we drew extended Bayesian skyline plots (eBSPs) with BEAST ver. 1.6.1 [41] using the population samples. The monomorphic loci of individual species, which cannot supply demographic information, were excluded. The best substitution model of each locus in every species was reevaluated with AIC and BIC scores using population samples (see Table S2). Ten million MCMC simulations were run to obtain better setting parameters for the priors and operators. Then 50 million MCMC simulations were performed with sampling every 1000 generations and the first 10% discarded as burn in to obtain eBSP results.

### Mismatch Analysis under a Spatial Expansion Model

Mismatch analysis was performed to evaluate the range expansions of five *Scutellaria* species under the spatial expansion model [58] using Arlequin var. 3.11 [59]. The infinite-island (demes) model with the assumption of constant population size (*θ* = *θ*_0_ = *θ*_1_) was assumed for the expectation, and each deme of a population exchanges a fraction *m* of migrants with other demes at time *τ* in this model [58]. The validity of the estimated expansion model was tested using the sum of squared deviations (SSD). Harpending’s [60] raggedness index *r*, which evaluates the smoothness of the observed mismatch curves, was also used to test demographic expansion. One thousand permutations were executed to evaluate the departures of observed data from expectations.

## Results

### Phylogenetic Relationships and Diversification Rate Assessment

Phylogenetic analysis of the set of 17 *Scutellaria* and other species illustrated close relationships among the seven Taiwanese species in the species tree, especially the group composed of *S. indica*, *S. tashiroi*, *S. austrotaiwanensis*, and *S. playfairii* (namely, the indica group; see Figure 2A) despite certain differences in the gene trees of a single locus (Figure S1). With the exception of the wide distribution of *S. indica* in Asia, the species of the indica group are endemic to Taiwan. The divergence times of these four species were short (79.8–204.1 Kya). Most interesting is that the American species *S. lateriflora* and inland Chinese species *S. sessilifolia* are epiphyletic to the indica group with divergence times of 274.6 Kya and 421.7 Kya, respectively, and another endemic Taiwanese species, *S. taiwanensis*, diverged with them at 472.2 Kya. The other two Taiwanese species, *S. barbata* and *S. taipeiensis*, split very recently (15.4 Kya) and are more deeply divergent with the other five Taiwanese species at 610.4 Kya. Despite the incomplete sampling of related species, this result provides evidence of various sources of Taiwanese species and recent and rapid speciation after colonization in Taiwan.

The late, rapid appearance of the LTT slopes in the Taiwanese species and endemic species also suggests a recent colonization or speciation of *Scutellaria* in Taiwan (Figure S2). Three LTT patterns, the density-dependent diversification (DDD), the constant rate of diversification (CRD), and the late burst of diversification (LBD) (Fig. 2B, see [61]), illustrated the basic hypotheses of diversification rates of species appearance by colonization and speciation. Based on the last 1000 postconvergence species trees devised using BEAST, the LTT analysis showed a delayed increase in the species accumulation rate in Taiwanese endemic *Scutellaria* species and suggested an LBD pattern of endemic species appearance in Taiwan, whereas the total Taiwanese *Scutellaria* species showed a constant rate of diversification pattern of gradual colonization/speciation in Taiwan (see Figure 2C). In addition to results of the LTT analysis, the test for heterogeneous diversification rates based on the topological analysis indicated significant among-lineage rate shift according to Colless’s imbalance index (*P* = 0.030), *M*_Π_ and *M*_Π_* (*P* = 0.035 and 0.044, respectively) and *M*_Σ_ and *M*_Σ_* (*P* = 0.026 and 0.033, respectively; Table S3). However, nodes A and B and other nodes did not shift diversification rate among descendant lineages according to Δ-statistics (see Figure 2A). Although the incomplete sampling of related species might interfere with this inference, the TSI-ERM model decreased the effect of sampling bias [55]. The finding of significant heterogeneous diversification rate in the whole tree without rate shift at any node reflects a gradual process of diversification rate change instead of punctuated rate shift [55].

### Topological Tests of the Origin Hypotheses

To test whether the Taiwan *Scutellaria* species rapidly diverged after the colonization of their common ancestor in Taiwan, we used the species tree (see Figure 2A) to test the origin hypotheses (see Figure 3). The hypotheses of a single origin for all Taiwanese species and the indica group and *S. taiwanensis* were all rejected by the AU, KH, and SH tests, which means that the Taiwanese *Scutellaria* species have at least three sources: one that derived *S. barbata* and *S. taipeiensis*, one that derived *S. taiwanensis*, and one that derived the indica group. The topological hypothesis of collapsed lineages of the indica group (see Figure 3B) was also rejected by the AU and KH tests despite not being rejected by the SH test (*P* = 0.486). The tree topology inferred from the pure-birth branching process (the Yule model) using BEAST was not rejected by the three tests (see Figure 3A), implying a gradual instead of a punctuated speciation process for the indica group in Taiwan.

### Biogeographic Inferences

**Figure 4:**
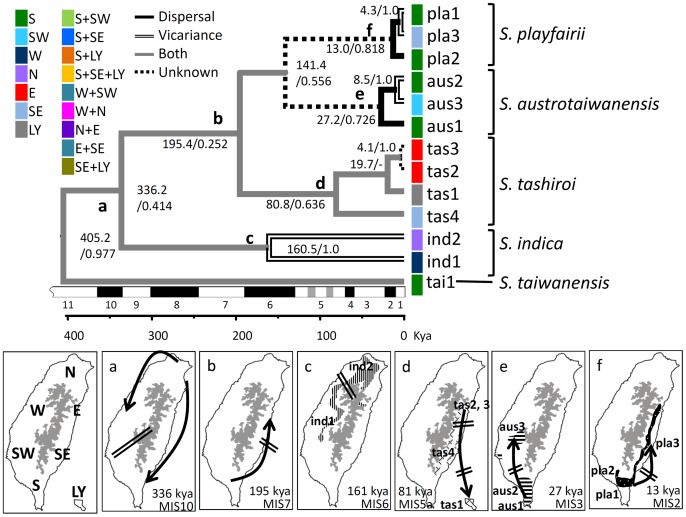
Graphical depiction of the ancestral distribution of populations of the indica group inferred with the statistical dispersal-vicariance analysis (S-DIVA) of the BEAST tree. Node labels are the divergence time (Kya) and the marginal probability of the node events; biogeographic events are indicated in different branch types; pie charts indicate the proportion of the ancestral ranges; and marine Isotope Stages (MIS) are indicated on the time scales. The pie chart with a black margin infers experienced extinction events. Biogeographic inferences of nodes a–f are presented in the lower panels. The arrow indicates the direction of dispersal, and the double lines indicate vicariance events. The distribution areas of extant populations of *S. indica*, *S. tashiroi*, *S. austrotaiwanensis*, and *S. playfairii* are marked in the panels c–f, respectively. The gray area in the middle of Taiwan indicates mountains with altitudes of >2000 m.

To understand the process of speciation and colonization of the rapidly divergent indica group, S-DIVA was performed. The ancestors of the indica group first colonized northern and eastern Taiwan around 336 Kya and then dispersed southward to western, southern, and southeast Taiwan. A vicariance event followed that has been inferred to have separated the southern and western ancestral populations (see Figure 4A). The northern and western lineages were ancestors of the extant populations of *S. indica*, which experienced another vicariance event at 161 Kya (see Figure 4C). The other lineage that colonized southern and southeastern Taiwan dispersed northward to eastern Taiwan at 195 Kya (see Figure 4B) and became the ancestor of *S. tashiroi*. The eastern and southeast populations of *S. tashiroi* then colonized southward to the Lanyu Islet, after which two vicariance events separated the eastern, southeast, and Lanyu populations at 81 Kya (see Figure 4D). Uncategorized events were identified for the divergence of the southern ancestral populations at 141 Kya, which originated *S. austrotaiwanensis* and *S. playfairii*. Two independent northward colonization events from the south to the southwest and southeast with later vicariances resulted in the present distribution of extant populations of *S. austrotaiwanensis* and *S. playfairii* at 27 Kya and 13 Kya, respectively (See Figure 4E and 4F). S-DIVA provides a clear biogeographic inference for the indica group in Taiwan.

### Genetic Diversity Estimation

Genetic variations within species and within populations were extremely low in species and population examination (Table 2 and S3). With the exception of two samples in population pla3 of *S. tashiroi* that were heterozygous in *CHS* (*h* = 9.09% and 2.94 for population pla3 and for *S. tashiroi*, respectively), the samples were homozygous. All samples of the five species were homozygous in locus *CAD*. No variation was discovered in 31 samples of *S. taiwanensis* at all loci. Among the indica group, *S. austrotaiwanensis* had the lowest diversity in nuclear loci and was monomorphic in chloroplast loci. The other two endemic species, *S. playfairii* and *S. tashiroi*, which sympatrically or parapatrically distributed in the eastern and southeastern Taiwan, had similar degrees of genetic diversity slightly higher than that of *S. austrotaiwanensis*. In contrast, the widely distributed *S. indica* had the highest genetic diversity (see Table 2). However, no variation was detected within populations of *S. indica* at all loci, whereas at least one locus was polymorphic at the population level in the other endemic species of the indica group (Table S4). These results indicate low genetic diversity of the indica group and suggest degrees of genetic differentiation between populations. In addition, positive but insignificant estimates of Tajima’s *D* and Fu’s *F*s statistics were made at all loci of every species, implying a subdivision of populations of these species, whereas the statistical insignificance suggests a failure to reject population size change or obviously structured populations.

**Table 2:** Genetic diversity of populations of *Scutellaria indica*, *S. austrotaiwanensis*, *S. playfairii*, *S. tashiroi*, and *S. taiwanensis*.

Locus	Species/Populations	*N*	*H*	*S*	*Hd* ± std	*π* ± std	*θ* ± std	Tajima’s *D*	*p*	Fu’s *F*	*p*
*CAD*	*S. austrotaiwanensis* * †	66	3	2	0.671±0.015	0.001±4e−5	3.7e−4±2.7e−4	1.915	0.967	2.024	0.864
*CAD*	*S. indica* †	21	2	6	0.514±0.046	0.003±2e−4	0.001±0.001	2.671	0.999	7.641	0.995
*CAD*	*S. playfairii* * †	83	3	2	0.545±0.025	0.001±4e−5	4e−4±3e−4	0.739	0.791	1.032	0.739
*CAD*	*S. tashiroi* * †	68	3	2	0.648±0.025	0.001±3e−5	4e−4±3e−4	2.111	0.979	2.203	0.890
*CHS*	*S. austrotaiwanensis* * †	132	2	1	0.413±0.034	0.001±4e−5	2e−4±2e−4	1.363	0.900	2.011	0.864
*CHS*	*S. indica* †	42	2	4	0.502±0.027	0.003±1e−4	0.001±0.001	2.735	0.997	6.824	0.989
*CHS*	*S. playfairii* * †	166	2	1	0.491±0.013	0.001±2e−5	2e−4±2e−4	1.868	0.961	2.554	0.908
*CHS*	*S. tashiroi* * †	136	6	4	0.652±0.033	0.002±1e−4	0.001±0.001	2.078	0.977	1.362	0.764
*ndh*F-*rpl*32	*S. indica* †	21	2	3	0.514±0.046	0.003±2e−4	0.001±0.001	2.234	0.991	4.389	0.975
*ndh*F-*rpl*32	*S. playfairii* * †	83	3	3	0.360±0.058	0.001±2e−4	0.001±0.001	0.184	0.636	1.328	0.791
*ndh*F-*rpl*32	*S. taiwanensis* †	31	2	1	0.452±0.063	0.001±1e−4	4e−4±4e−4	1.240	0.884	1.459	0.824
*ndh*F-*rpl*32	*S. tashiroi* * †	68	5	5	0.717±0.038	0.003±3e−4	0.002±0.001	1.763	0.963	2.215	0.852
*rpl*32-*trn*L	*S. indica* †	21	2	6	0.514±0.046	0.005±5e−4	0.003±0.001	2.671	0.999	7.641	0.995
*rpl*32-*trn*L	*S. playfairii* * †	83	3	6	0.626±0.023	0.005±1e−4	0.002±0.001	3.018	0.999	8.057	0.986
*rpl*32-*trn*L	*S. tashiroi* * †	68	2	3	0.504±0.015	0.003±8e−5	0.001±0.001	2.728	0.996	6.085	0.982

Tajima’s *D* and Fu’s *F*s were estimated based on 1000-repeat coalescent simulations in consideration of recombination.

*N* is the number of sequences; *H* is the number of haplotypes; *Hd* is the haplotype diversity; *π* and *θ* are the nucleotide diversity estimated by pairwise differences and number of segregating sites, respectively.

Chloroplast *mat*K, which is monomorphic in every species, is excluded.

*S. taiwanensis* (*N* = 31), which is monomorphic at loci *CAD*, *CHS*, and *rpl*32-*trm*L, and *S. austrotaiwanensis*, which is monomorphic at loci *ndh*F-*rpl*32 and *rpl*32-*trm*L, are not shown.

* endemic to Taiwan;

† members of the indica group.

Detailed information about the genetic diversity of each sample population is given in Table S4.

### Population Structure

Given the inference of population subdivision made through positive estimates of Tajima’s *D* and Fu’s *F*s and the vicariance inferences of all species of the indica group made with S-DIVA, AMOVA was performed to examine whether the extant populations of Taiwanese *Scutellaria* species are structured (Table 3). Significant genetic differentiation was examined between species at all five loci (*P*<0.05) in both sets of comparisons (five-species including *S. taiwanensis* and the indica group only), but *CAD* (Φ_CT_ = 0.060, *P* = 0.0899) was used for four-species examination. Significant population structures within species were also detected in every species at most loci except the intraspecies-monomorphic *mat*K. The proportion of genetic variation was mostly contributed at this level (among populations within species), especially at the nuclear markers (except *ndh*F-*rpl*32). The high Φ_SC_ and Φ_ST_ of most loci (except *mat*K) and significant deviation from random variation (*P*<0.05) indicated that the extant populations of the indica group are differentiated between one other. In addition, the higher components of variation among populations within species compared with those within populations also suggest structured populations in every species that we examined.

**Table 3:** Summary of two comparisons for analysis of variance (AMOVA) using two nuclear loci and three chloroplast loci.

Locus	Source of variation	df	S.S.	Var. Comp.	% Var.	Φ	P
Five species (the indica group + *S. taiwanensis*)							
*CAD*	Among species	4	47.590	0.037	7.32	0.073	0.0332
	Among populations within species	8	62.309	0.420	83.89	0.905	<0.0001
	Within populations	256	11.264	0.044	8.79	0.912	<0.0001
*CHS*	Among species	4	114.001	0.115	23.01	0.23	0.0049
	Among populations within species	8	103.827	0.351	70.13	0.911	<0.0001
	Within populations	525	18.030	0.034	6.86	0.931	<0.0001
*mat*K	Among species	4	44.368	0.217	100	1	0.002
	Among populations within species	8	0	0	0	0	1
	Within populations	256	0	0	0	1	<0.0001
*ndhF-rpl32*	Among species	4	60.334	0.220	45.78	0.458	<0.0001
	Among populations within species	8	23.822	0.156	32.38	0.597	<0.0001
	Within populations	256	26.895	0.105	21.84	0.782	<0.0001
*rpl*32*-trn*L	Among species	4	58.222	0.178	38.84	0.388	0.002
	Among populations within species	8	33.839	0.226	49.37	0.807	<0.0001
	Within populations	256	13.846	0.054	11.79	0.882	<0.0001
Four species (the indica group only)							
*CAD*	Among species	3	32.372	0.030	6.03	0.060	0.0899
	Among populations within species	8	62.309	0.420	84.00	0.894	<0.0001
	Within populations	226	11.264	0.050	9.97	0.900	<0.0001
*CHS*	Among species	3	82.722	0.110	22.00	0.220	0.0059
	Among populations within species	8	103.827	0.351	70.22	0.900	<0.0001
	Within populations	464	18.030	0.039	7.78	0.922	<0.0001
*mat*K	Among species	3	19.147	0.113	100	1	0.0108
	Among populations within species	8	0	0	0	0	1
	Within populations	226	0	0	0	1	<0.0001
*ndhF-rpl32*	Among species	3	49.691	0.232	48.56	0.4856	0.0010
	Among populations within species	8	23.822	0.157	32.80	0.6376	<0.0001
	Within populations	226	20.121	0.089	18.64	0.8136	<0.0001
*rpl*32*-trn*L	Among species	3	41.016	0.155	35.00	0.3500	0.0127
	Among populations within species	8	33.839	0.226	51.14	0.7868	<0.0001
	Within populations	226	13.846	0.061	13.86	0.8614	<0.0001

The indica group indicates *S. indica*, *S. austrotaiwanensis*, *S. playfairii*, and *S. tashiroi*.

### Historical Demographics

**Figure 5:**
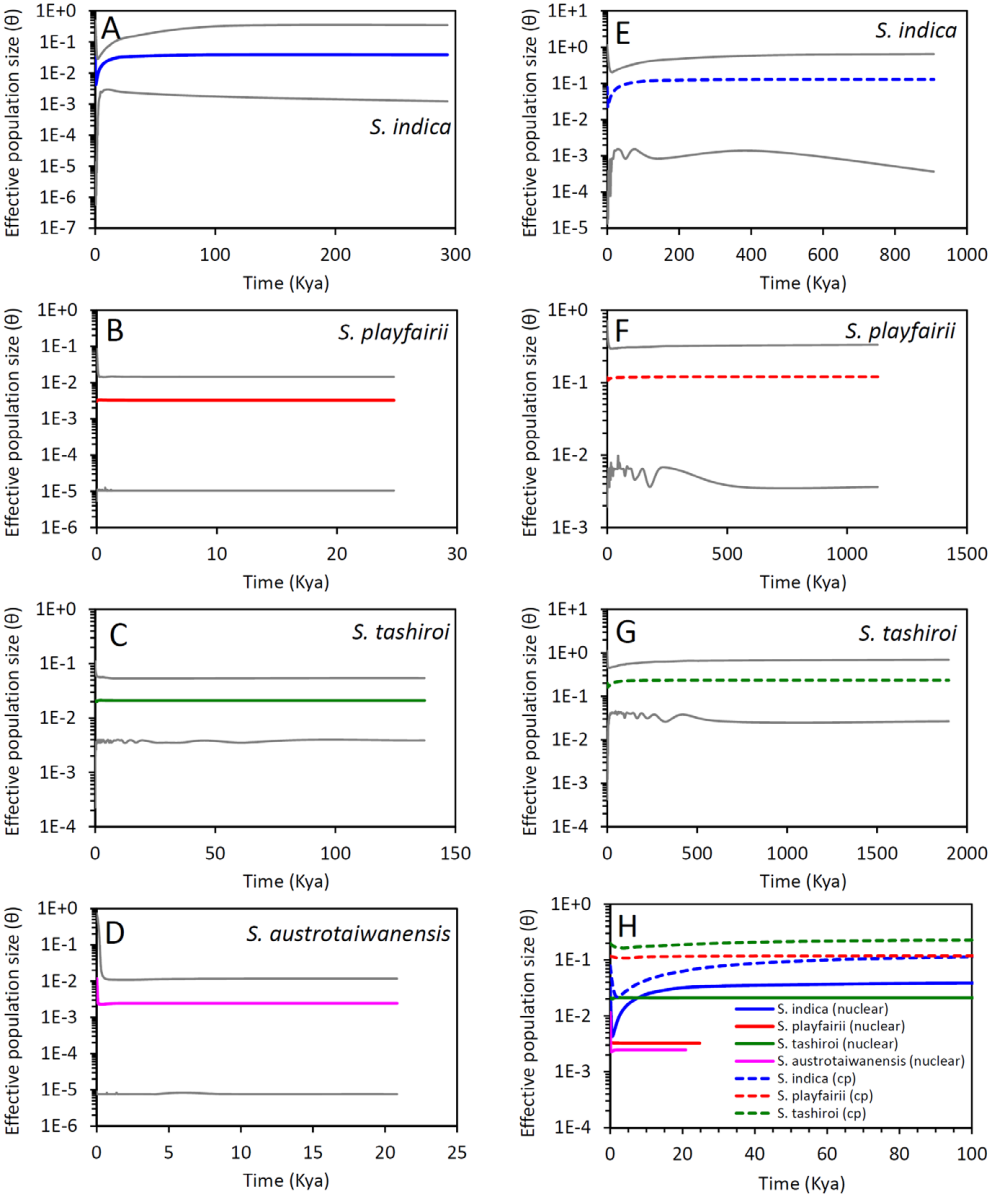
Extended Bayesian skyline plots (eBSPs) of members of the indica group in Taiwan. A–D are eBSPs inferred from nuclear loci, and E–G are eBSPs of chloroplast (cp) loci. Figure H is a close look at the eBSPs of all species since 100 Kya. Solid and dotted lines indicate the median curves of the eBSPs inferred from nuclear and cpDNA, respectively. The gray curves are the 95% highest posterior density intervals of the eBSPs. Note that the very recent population size increase of *S. austrotaiwanensis* was inferred from nuclear loci, and a very recent recovery of the effective population sizes after bottlenecks was inferred in *S. indica*. Population sizes of both *S. playfairii* and *S. tashiroi* are constant in coalescent processes. The chloroplast eBSPs could not be inferred in *S. austrotaiwanensis* because of monomorphism.

Demographic changes of the indica group were inferred based on eBSP analysis, which revealed long-term constant population sizes in these five species until thousands of years ago. *Scutellaria indica* revealed a serious bottleneck event beginning at approximately 20 Kya in the nuclear inference or earlier in the cpDNA inference (Figure 5). Both nuclear and cpDNA inferences of demographic history showed a very recent population-size recovery decades or hundreds of years ago. Differences in the inferences of the timing of the bottlenecks made using nuclear and cpDNA probably resulted from heterogeneous rates of lineage sorting of genomes, which are also reflected in differences in population size inferences. In contrast to the bottleneck of *S. indica*, that of *S. austrotaiwanensis* was inferred from evidence of a long-term constant population size followed by a very recent population size increase demonstrated by nuclear loci, although the wide range in the confidence interval caused by small variations might mislead the inference. The chloroplast eBSP was unable to construct *S. austrotaiwanensis* because of monomorphism in all chloroplast loci. In addition to demographic fluctuations in *S. indica* and *S. austrotaiwanensis*, permanent stable demography was found in the other two species of the indica group in both nuclear and cpDNA inferences (see Figure 5).

For understanding better whether these Taiwanese *Scutellaria* species underwent historical range expansion, the SSD statistic and raggedness index were used to test the goodness of fit of the observed mismatch distributions to the expectations under the spatial expansion model using four polymorphic loci. Significant SSD values of four loci were taken as evidences of deviation from the estimated demographic model of spatial expansion in populations of *S. indica.* Significant SSD was also estimated for the nuclear loci of *S. playfairii* and *CHS* of *S. austrotaiwanensis* and the chloroplast locus *rpl*32-*trn*L of *S. tashiroi* and *ndh*F-*rpl*32 of *S. taiwanensis*. The significant values indicated that these loci cannot reflect the patterns of spatial expansion of these species. In contrast, the nuclear *CAD* of *S. austrotaiwanensis* and *S. tashiroi*, *CHS* of *S. tashiroi* as well as chloroplast *ndh*F-*rpl*32 of *S. playfairii* and *S. tashiroi* and *rpl*32-*trn*L of *S. playfairii* failed to reject the expectation of spatial expansion according to both SSD and raggedness indices. We also combined four members of the indica group for a mismatch analysis and obtained results of a failed rejection of range expansion in estimations of nuclear and chloroplast loci according to both SSD and raggedness indices (Table 4), which supports the inference of multiple dispersals suggested by S-DIVA.

**Table 4:** Summary of the test of spatial expansion model through the mismatch analyses.

	Parameter	indica group	*S. indica* †	*S. tashiroi* † ‡	*S. playfairii* † ‡	*S. austrotaiwanensis* † ‡	*S. taiwanensis* ‡
*CHS*	τ (95% CI)	**4.9 (0.478–114.701)**	4.6 (0.000–66.882)	**2.2 (0.394–5.715)**	0.7 (0.405–0.922)	0.6 (0.374–0.763)	–
	t (95% CI)	**0.215(0.021–5.031)**	0.202**(**0.000**–**2.933**)**	**0.096(0.017–0.251)**	0.031**(**0.018**–**0.040**)**	0.026**(**0.016**–**0.033**)**	–
	θ (95% CI)	**4.157 (0.001–10.094)**	0.001 (0.001–1.049)	**0.545 (0.001–1.541)**	0.001 (0.001–0.421)	0.004 (0.001–0.351)	–
	M (95% CI)	**2.233 (0.451–99999)**	1.271 (0.000–5.866)	**1.899 (0.597–99999)**	99999 (6.037–99999)	99999 (3.168–99999)	–
	SSD	**0.011**	0.173*	**0.019**	0.02*	0.008*	–
	r	**0.033**	0.752	**0.088**	0.241*	0.201*	–
*CAD*	τ (95% CI)	**5.0 (2.133–14.223)**	6.7 (0.000–50.500)	**1.3 (0.449–2.144)**	0.8 (0.423–1.106)	**0.6** (**0.399–4.211**)	–
	t (95% CI)	**0.143(0.061–0.408)**	0.192**(**0.000**–**1.449**)**	**0.037(0.013–0.062)**	0.023**(**0.012**–**0.032**)**	**0.017(0.011–0.121)**	–
	θ (95% CI)	**3.083 (0.001–7.201)**	0.001 (0.001–1.137)	**0.012 (0.001–0.921)**	0.006 (0.001–0.377)	**1.295** (**0.001–3.151**)	–
	M (95% CI)	**3.565 (0.761–14.919)**	1.28 (0.000–4.870)	**15.871 (1.675–99999)**	99999 (3.177–99999)	**99999** (**0.812–99999**)	–
	SSD	**0.019**	0.196*	**0.008**	0.031*	**0.004**	–
	r	**0.033**	0.765	**0.092**	0.219*	**0.035**	–
*ndh-rpl*32	τ (95% CI)	**9.756 (0.789–20.893)**	3.488 (0.000–85.625)	**11.464 (0.430–25.197)**	**11.858** (**0.000–170.500**)	–	0.665 (0.307–1.342)
	t (95% CI)	**4.302(0.348–9.212)**	1.538**(**0.000**–**37.754**)**	**5.055(0.190–11.110)**	**5.228(0.000–75.176)**	–	0.293**(**0.135**–**0.592**)**
	θ (95% CI)	**2.555 (0.001–6.080)**	0.001 (0.001–1.647)	**2.172 (0.001–3.843)**	**0.001** (**0.001–0.570**)	–	0.004 (0.001–0.596)
	M (95% CI)	**1.448 (0.383–99999)**	1.442 (0.000–99999)	**0.377 (0.243–99999)**	**0.624** (**0.000–2.556**)	–	99999 (0.390–99999)
	SSD	**0.045**	0.169*	**0.02**	**0.094**	–	0.013*
	r	**0.114**	0.765	**0.061**	**0.646**	–	0.213
*rpl*32*-trn*L	τ (95% CI)	**5.607 (2.595–9.123)**	14.922 (0.000–85.813)	3.488 (0.306–51.131)	**5.536** (**2.036–11.850**)	–	–
	t (95% CI)	**2.425(1.122–3.946)**	6.454**(**0.000**–**37.116**)**	1.509**(**0.132**–**22.115**)**	**2.394(0.881–**−**5.125)**	–	–
	θ (95% CI)	**0.106 (0.001–1.651)**	0.001 (0.001–0.865)	0.001 (0.001–1.249)	**0.403** (**0.001–1.856**)	–	–
	M (95% CI)	**2.977 (0.780–10.551)**	1.21 (0.000–4.957)	1.371 (0.093–99999)	**1.5** (**0.297–6.441**)	–	–
	SSD	**0.026**	0.218*	0.162*	**0.088**	–	–
	r	**0.139**	0.765	0.754	**0.273**	–	–
*mat*K	τ (95% CI)	**3.5 (0.000–95.739)**	–	–	–	–	–
	t (95% CI)	**1.195(0.000–32.698)**	–	–	–	–	–
	θ (95% CI)	**0.001 (0.001–0.729)**	–	–	–	–	–
	M (95% CI)	**0.217 (0.000–99999)**	–	–	–	–	–
	SSD	**0.016**	–	–	–	–	–
	r	**0.761**	–	–	–	–	–

Indices of monomorphic loci within species are shown in “–.” CI: confidence interval, α = 0.05; SSD: sum of squared deviations; r: raggedness index; τ: unscaled expansion time; t: scaled expansion time (unit: Mya); θ: unscaled population size.

* *P*<0.05;

** *P*<0.01;

*** *P*<0.00001 for the sum of squared deviations and raggedness index that reject the null hypothesis of expectation under a sudden expansion model.

† members of the indica group;

‡ endemic to Taiwan.

The time at which the range expansion events took place was dated using the expression *t* = *τ*/2*µk*, where the *τ* is the estimated number of generations after expansion, *μ* is the mutation rate per site per generation, and *k* is the sequence length. Mutation rates of 1.5% and 0.2% per site per million years were used for nuclear and chloroplast loci, respectively. A relatively short spatial expansion time at 0.017 Mya (95% confidence interval [95% CI]: 0.011–0.121 Mya) was estimated for *S. austrotaiwanensis* with *CAD*, and earlier expansion events occurred for *S. tashiroi* at 0.037 Mya (95% CI: 0.013–0.062 Mya) according to *CAD* or 0.096 Mya (95% CI: 0.017–0.251 Mya) according to *CHS.* Owing to its slower substitution rates, cpDNA may reflect events in the distant past. The spatial expansion time was estimated at 2.394 Mya (95% CI: 0.881∼5.125 Mya) with *rpl*32-*trn*F or 5.228 Mya (95% CI: 0–75.176 Mya) with *ndh*F-*rpl*32 for *S. playfairii* and 5.055 Kya (95% CI: 0.190–11.110 Mya) for *S. tashiroi*. These results suggest that the relatively recent spatial expansions of *S. austrotaiwanensis* and past expansions of *S. playfairii* and *S. tashiroi* were probably gradual events of long duration, a characteristic also reflected in the wide-ranging 95% CI associated with both nuclear and chloroplast markers. In addition, the spatial expansion time of whole members of the indica group was inferred as 0.215 Mya (95% CI: 0.021–5.031 Mya), 0.143 Mya (95% CI: 0.061–0.408 Mya), 4.302 Mya (95% CI: 0.348–9.212 Mya), 2.425 Mya (95% CI: 1.122–3.946 Mya), and 1.195 Mya (95% CI: 0–32.698 Mya) according to *CHS*, *CAD*, *ndh*F-*rpl*32, *rpl*32-*trn*F, and *mat*K, respectively. Notably, the estimated expansion times were longer than the coalescent time estimated by the species trees (see Figures 2 and 4), especially for the chloroplast estimations, which could be explained by the slower evolutionary rates of the plastid genomes of plants and different coalescent rates of loci.

## Discussion

### Multiple Origins of Highly Endemic Scutellaria Species in Taiwan

Species on continental islands are mainly sourced from neighboring continents and local speciation. Taiwan is situated in southeastern Asia and is a component of the west Pacific Island arc. The enormous drop in sea level during the Pleistocene glacials formed land bridges that connected Taiwan, mainland Asia, and the Ryukyu Archipelago and accelerated organism colonization of Taiwan (e.g., weasels [62], freshwater fish [63], and damselfly [64]) and movement from Taiwan (e.g., *Ophiorrhiza japonica* [65]). Accordingly, all of the Taiwanese skullcaps are morphologically close and might be of single origin [29]. However, the results of phylogenetic and topological analyses (AU, KH, and SH tests) indicate at least three incidences of origination of Taiwanese endemic *Scutellaria* species (Figure 3) with resulting local speciation events between ∼0.2 Mya (the indica group) and 0.02 Mya (*S. taipeiensis*) (see Figure 2A). The rejection of collapsed phylogenetic topology at the common ancestor of the indica group by the AU and KH tests (see Figure 3B) suggests that the speciation of endemic *S. austrotaiwanensis*, *S. playfairii*, and *S. tashiroi* did not display temporal bursts of diversification [66], [67] but a gradual process of speciation. In other words, the hypothesis that a single radiation event could explain the high endemism of Taiwanese *Scutellaria* via various types of adaptation among species [67] is untenable. This rejection was also evidenced by the failure to detect diversification rate shifts at all nodes of the Taiwanese lineages according to Δ-statistics (see Figure 2A) and insignificant tail probabilities of asymmetric diversification rate indices of Taiwanese *Scutellaria* species (see Table S3). The steep and delayed increase in species accumulation rate in both clusters of the Taiwanese species and endemic species revealed in LTT curves (see Figure S2) indicate very recent species appearance (i.e., very recent colonization and speciation) of *Scutellaria* species in Taiwan, especially in endemic species that follow an LBD pattern (see Figure 2C). The rapid local speciation was probably involved in local adaptation and geographic isolation in the ragged topography and high Central Mountain Range of Taiwan Island, which provide diverse habitats and geographic barriers for distant populations.

With the exception of *S. taiwanensis*, the endemic *Scutellaria* species (i.e., *S. taipeiensis* and three endemic species of the indica group) were grouped with a corresponding widespread Asian species. Divergence times between endemic *S. playfairii* and *S. austrotaiwanensis* and between *S. tashiroi* and the most recent common ancestor (MRCA) of *S. playfairii* and *S. austrotaiwanensis* were ∼79.8 Kya and ∼121.6 Kya, respectively. These times roughly correspond to the interglacial Marine Isotope Stage (MIS) 5a and MIS5e, respectively. The divergence time between the widely distributed *S. indica* and endemic species of the indica group was ∼204.1 Kya, roughly the period of the interglacial MIS7. Notably, these species display geographically parapatric distribution, and *S. indica* is nearly allopatric with the endemic members of the indica group (see Figure 1A). Although the Taiwanese topography is ragged, the vicariance hypothesis of speciation is untenable because the divergent times of species occurred much later than the geographic events (e.g., orogenesis). In contrast, species divergence during the warm interglacial periods was probably caused by long-distance dispersal followed by geographic isolation [68], [69]. Such scenarios were also proved with S-DIVA (see Figure 4).

*Scutellaria taipeiensis*, which has the shortest coalescent history, diverged from the widespread Asian *S. barbata* at ∼15.4 Kya (see Figure 2A), or roughly during the last glacial maximum (LGM, the MIS2). Indeed, seed dispersal is presumably short if it relies on capsule burst. Long-distance dispersal may be difficult and occur only during flooding or via rivers [27], [70], but the hydrology of Taiwan was highly constrained during glacial periods. The extant population of *S. taipeiensis* is found only at low altitudes in northern Taiwan. The cold and dry climate during MIS2 might have played a role as the limiting factor for the distribution of common ancestors of *S. taipeiensis* and *S. barbata*. Populations that retreated into isolated refugia might have finally diverged from the others. Range expansion from refugia at the next warm interglacial (MIS1) explains the sympatric distribution of extant populations of *S. barbata* and *S. taipeiensis* in northern Taiwan.

### Biogeographic Patterns of the Indica Group

The ragged topography of Taiwan provided multiple habitat choices for founders of species. According to the S-DIVA inference, founders of common ancestors of the indica group first colonized northern and northeastern Taiwan at approximately 336.2 Kya–the end of glacial stage MIS10 (see Figure 4). During the glacial periods, seabeds of the Taiwan Strait emerged owing to sea-level regression, connecting northern Taiwan and mainland Asia. Therefore, the seeds of founders likely colonized Taiwan through the paleo-river system on the emerged seabed of the northern Taiwan Strait. The ancestors of the indica group species, which grew in wet habitats, could have entered Taiwan via the water system of the alternate path of the Paleo-Minjiang River (APPM) to the northern part of Taiwan, one staying in the northwest part and another moving east (see Figure 1B), and consequently migrated to the southwest and southeast, respectively. However, the Penghu submarine canyon off of southwestern Taiwan separated southern Taiwan from mainland Asia [71] and might also have hindered southern colonizers into Taiwan. Similar effects of geographic barrier by the Paleo-Minjiang River can be seen in damselfly [64], freshwater crabs [72], and landlocked shrimp [73]. The warm interglacial period that followed (MIS9) accelerated southward dispersal throughout both the eastern and the western sides of the Central Mountain Range. The western lineage (descendants of node C, the extant populations of *S. indica*; see Figure 4) experienced a vicariance event during glacial stage MIS6 (∼160.5 Kya). The eastern lineage (descendants of node B; see Figure 4) further evolved gradually as three endemic species through serial processes of dispersal and vicariance. The southern ancestral population colonized northward through eastern Taiwan during interglacial MIS7 (∼195.4 Kya) and became isolated from its southern ancestors. The eastern population further speciated as *S. tashiroi* and dispersed southward again at interglacial MIS5a (∼80.8 Kya) with two vicariance events following. In contrast, the southern population somehow separated into two lineages (*S. austrotaiwanensis* and *S. playfairii*) and dispersed northward through the western and eastern Central Mountain Range during MIS3 (27.2 Kya) and the late MIS2 (13.0 Kya), respectively. Then, both dispersed northward and pre-species of *S. austrotaiwanensis* and *S. playfairii* separated from their southern ancestors and speciated further (see Figure 4).

S-DIVA illustrated a biogeographic pattern of dispersal/colonization during the warm interglacials with isolation following. Warm and wet weather during the interglacials could have led to greater water flow in rivers and more pollinators and may have prompted long-distance dispersal followed by colonization or vicariance. Furthermore, dispersal (colonization) promotes the establishment of novel niches and speciation [74]. Although an exceptional diversification rate, which is an essential condition of adaptive radiation [67], is absent in endemic *Scutellaria*, the temporally differential distribution consequence of niche allocation accelerated the speciation rate of *Scutellaria* in Taiwan. The flowering time of the Taiwanese *Scutellaria* is predominantly during summer [30], whereas *S. indica* has a longer and earlier flowering time (September to May) than that of other endemic members of the indica group (June to December) [30]. *Scutellaria indica* is distributed in northern and western Taiwan, in contrast to the southern and eastern distribution of the other endemic indica group members. Furthermore, *S. tashiroi* prefers more open rocky cliffs, whereas *S. austrotaiwanensis* and *S. playfairii* prefer slightly shaded environments (personal observation). Such niche allocation of the indica group could explain their high endemism and rapid speciation rate after colonization in Taiwan.

### Demographic History of the Indica Group

In addition to elucidating the dispersal and fragmentation of populations of the indica group in Taiwan, we also found permanent constant population sizes in members of the indica group according to nonsignificant *P* values of Tajima’s *D* and Fu’s *F*s test using coalescent simulations (see Table 2). This finding is also evidenced by the past long-term stable populations determined by eBSP analysis, with the exception of the recent bottleneck event of *S. indica* and the very recent population size increase of *S. austrotaiwanensis* (see Figure 5). The beginning of population size decrease in Taiwanese *S. indica* is roughly consistent with the period of the LGM that majorly affected Taiwanese vegetation between 21 and 15.8 Kya [75]. The average temperature of the LGM was roughly 5°C lower than that of the present [76], and the drier climate altered the vegetation distribution during the LGM such that drought-enduring herbs–e.g., the *Artemisia*, *Apiaceae*, and *Poaceae*–dominated the island vegetation [75]. The colder and drier climate of the LGM might have forced populations of *S. indica* to retreat to refugia as small populations or altered the reproductive strategy to cleistogamy [24], resulting in severe population size decrease (see Figure 5). In contrast, the population sizes of the other endemic members of the indica group did not decrease during the LGM, likely because the speciation times of these endemic species were too short to accumulate much genetic variation (see Table 2), i.e. insufficient duration for coalescence [77], and thus, were genetically insensitive to demographic fluctuation (e.g., [78]).

Although the population sizes of endemic indica-group members were permanently constant, the spatial expansion model was not rejected in *S. austrotaiwanensis* and *S. tashiroi* according to nuclear loci or in *S. playfairii* and *S. tashiroi* according to cpDNA markers under mismatch analysis (see Table 4). These results indicate that although no lineage expansion occurred in the coalescent process, but the extant lineages migrated and expanded geographically. Differing evolutionary rates of nuclear and plastid genomes result in different coalescent estimates (e.g., expansion time and demographic history). In other words, different rates of lineage sorting of various markers might result in different inferences of range expansion and demographic dynamics (see Table 4), which helps to infer the series time of range expansion. The higher evolutionary rate of nuclear DNA implies a shorter coalescent history of range expansion of *S. austrotaiwanensis*, whereas the lower evolutionary rate of cpDNA reflects a long-term spatial expansion of *S. playfairii*. *Scutellaria tashiroi* might have experienced a permanent spatial expansion after speciation for which the spatial expansion model is rejected by neither nuclear loci nor cpDNA *ndh*F-*trn*L32. Such inferences of range expansion are not only consistent with the inferences from S-DIVA but also reflect the various demographics in maternal and biparental inheritances–i.e., different dispersibility of seeds (reflected in both nuclear and cpDNA) and pollens (reflected in cpDNA only). Seed dispersal of *Scutellaria* is often mediated by gravity or water flow, which is affected by topography (e.g., river systems), whereas the pollination of *Scutellaria* is mediated by small Hymenoptera insects with short-distance pollen flow [35], [28]. The distance of pollen flow is presumably shorter than that of seed dispersal [27], [28], and therefore the migrants inferred by nuclear and plastid loci that effectively counteracted the drift effect could be different and lead to inconsistent demographic inferences.

### Conclusions

Our study examined the genetic diversity and historical demographic change among Taiwanese skullcaps (*Scutellaria*). The high endemism and similarity in these species make them suitable examples of the effects of glacial-interglacial change on biodiversity in recently evolved endemic species on a continental island. The results imply that the original Taiwanese *Scutellaria* species evolved at least three times instead of a single time with radiation. Our finding also thoroughly demonstrates continual divergence in Taiwanese *Scutellaria* species and their close phylogenetic relationships. A delayed increase in LTT curve and recent divergence time indicate recent colonization and local adaptation for the endemic species. One of the Taiwanese clades, the indica group, diverged recently, and most members are distributed sympatrically or parapatrically. During the glacial period MIS10 (336 Kya), during which the seabed of the Taiwan Strait emerged, the common ancestor of the indica group may have colonized northwestern and northeastern Taiwan via the Paleo-Minjiang River and its alternate paths, respectively. Subsequently, warm and wet interglacials facilitated long-distance dispersal via pollen flow through increased numbers of pollinators or seed transport to colonize novel habitats via water flow; cold and dry glacials led populations to retreat into refugia and resulted in fragmentation. Repeated dispersal and isolation diversified populations or species of the indica group. The demography of *S. indica*, which has relatively high genetic diversity compared with that of other members of indica group, was more sensitive to climate change–i.e., the bottleneck at LGM. In contrast, the endemic species of the indica group revealed higher dispersibility for range expansion in coalescent processes but constant population size through time. The newly derived species have not accumulated enough genetic variation yet and present difficulty in reflecting genetic loss through population decline, but low variation could be irrelevant to the estimation of migration. Based on this case study, we suggest that the diversification of continental island herbs might be related to the sources of adjacent areas and past climatic and geographic changes. The results of this study also implied that island species can rapidly fill open niches caused by cyclic glacials/interglacials via rapid diversification through repeated dispersal and vicariance.

## Supporting Information

Figure S1 **Neighbor-joining (NJ) and Bayesian inference (BI) trees of** ***Scutellaria*** **species reconstructed by each of five loci.** Species marked in red and red bold are distributed in Taiwan and endemic to Taiwan, respectively. Values indicated in the nodes are the bootstrap values and posterior probabilities for supporting the grouping of lineages in NJ trees and BI trees, respectively.
(DOCX)

Figure S2 **Comparison of Lineage-Through-Time (LTT) plots, showing the CRD pattern of all Taiwan** ***Scutellaria*** **species (black curves) and the LBD pattern of the endemic** ***Scutellaria*** **species (gray curves) based on 1000 postconvergence Bayesian trees.**
(DOCX)

Table S1 List of Scutellaria species used in the phylogenetic analysis.
(DOCX)

Table S2 Best substitution models for the five loci used in the Bayesian analyses.
(DOCX)

Table S3 Tail probabilities of asymmetric values for the among-lineage diversification rate variation in the phylogenetic topologies reconstructed from total samples and Taiwanese samples, respectively, inferred from the species tree (BEAST).
(DOCX)

Table S4 Genetic diversity of populations of *Scutellaria* species in Taiwan estimated using four polymorphic loci. The monomorphic *mat*K in every population is not shown.
(DOCX)
