# Supplementary material for: Diversification, Biogeographic Pattern, and Demographic History of Taiwanese Scutellaria Species Inferred from Nuclear and Chloroplast DNA
Source: PLoS One. 2012 Nov 30;7(11):e50844. doi: 10.1371/journal.pone.0050844 (PMC3511331; doi:10.1371/journal.pone.0050844)
Supplement: Figure S1 — Neighbor-joining (NJ) and Bayesian inference (BI) trees of Scutellaria species reconstructed by each of five loci. Species marked in red and red bold are distributed in Taiwan and endemic to Taiwan, respectively. Values indicated in the nodes are the bootstrap values and posterior probabilities for supporting the grouping of lineages in NJ trees and BI trees, respectively. (DOCX) [file pone.0050844.s001.docx]

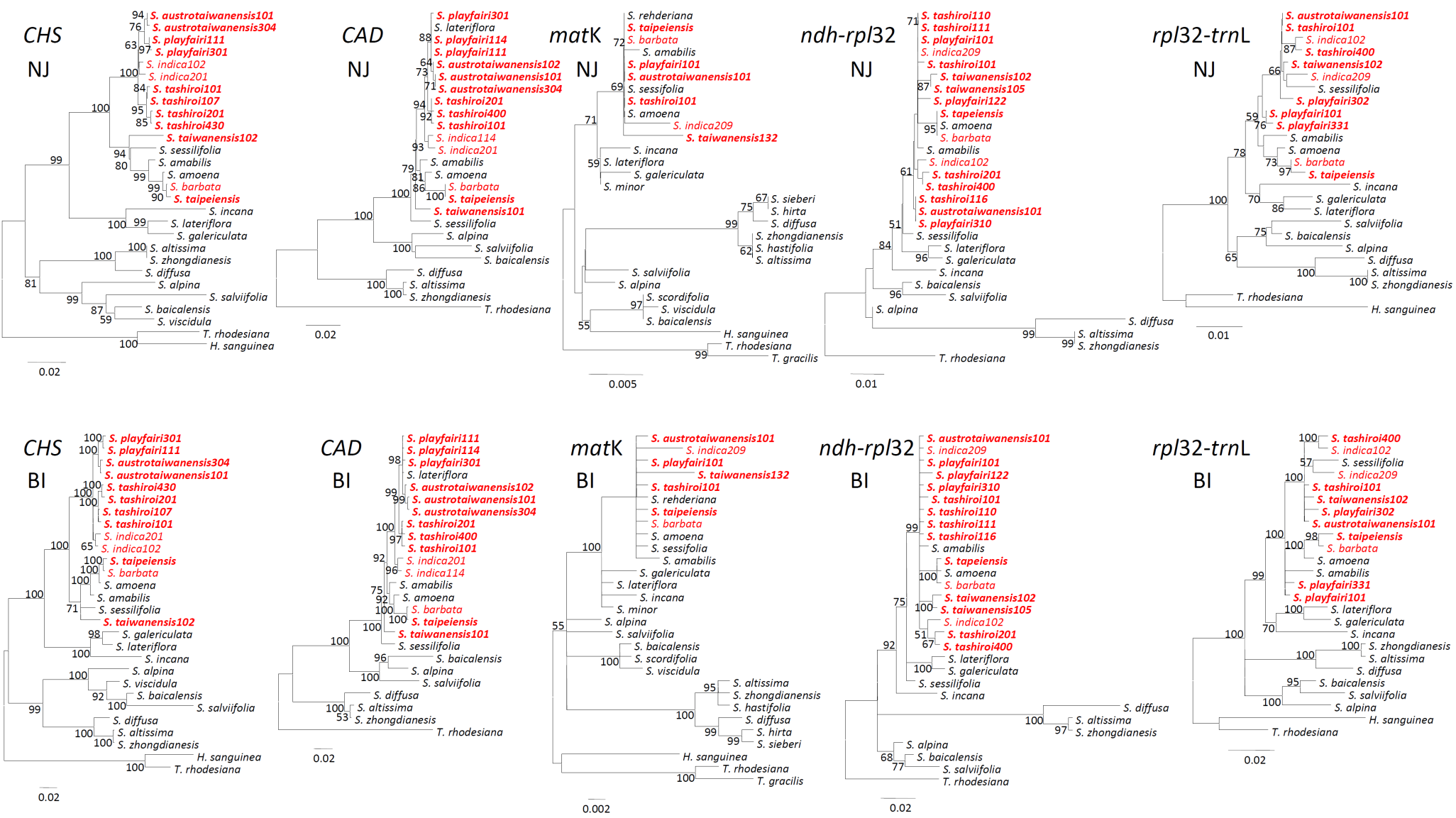


**Figure S1** Neighbor-joining (NJ) and Bayesian inference (BI) trees of *Scutellaria* species reconstructed by each of five loci. Species marked in red and red bold are distributed in Taiwan and endemic to Taiwan, respectively. Values indicated in the nodes are the bootstrap values and posterior probabilities for supporting the grouping of lineages in NJ trees and BI trees, respectively.
