# Supplementary material for: Diversification, Biogeographic Pattern, and Demographic History of Taiwanese Scutellaria Species Inferred from Nuclear and Chloroplast DNA
Source: PLoS One. 2012 Nov 30;7(11):e50844. doi: 10.1371/journal.pone.0050844 (PMC3511331; doi:10.1371/journal.pone.0050844)
Supplement: Figure S2 — Comparison of Lineage-Through-Time (LTT) plots, showing the CRD pattern of all Taiwan Scutellaria species (black curves) and the LBD pattern of the endemic Scutellaria species (gray curves) based on 1000 postconvergence Bayesian trees. (DOCX) [file pone.0050844.s002.docx]

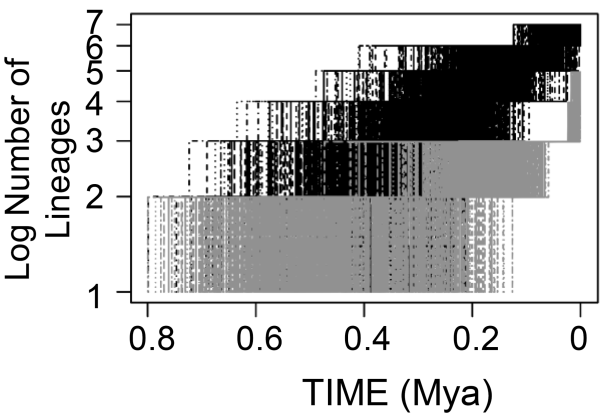


**Figure S2** Comparison of LTT plots showing the CRD pattern of all Taiwan *Scutellaria* species (black curves) and the LBD pattern of the endemic *Scutellaria* species (gray curves) based on 1000 postconvergence Bayesian trees.
