# Supplementary material for: Diversification, Biogeographic Pattern, and Demographic History of Taiwanese Scutellaria Species Inferred from Nuclear and Chloroplast DNA
Source: PLoS One. 2012 Nov 30;7(11):e50844. doi: 10.1371/journal.pone.0050844 (PMC3511331; doi:10.1371/journal.pone.0050844)
Supplement: Table S1 — List of Scutellaria species used in the phylogenetic analysis. (DOCX) [file pone.0050844.s003.docx]

**Table S1** List of *Scutellaria* species used in the phylogenetic analysis.

| Species | Distribution | Sample source |
| --- | --- | --- |
| *S. austrotaiwanensis* | Taiwan | Hengchun peninsula, Lilungshan, and Nanhua, Taiwan |
| *S. indica* | Taiwan | Awanda and Wulai, Taiwan |
| *S. playfairii* | Taiwan | Dahan trail, Wutai, and Wulu, Taiwan |
| *S. tashiroi* | Taiwan | Lanyu Island, Mugumuyu, Taroko, and Wulu, Taiwan |
| *S. taiwanensis* | Taiwan | Jin-Shui Camp, Taiwan |
| *S. barbata* | Taiwan | Yonghe Dist., Taipei, Taiwan |
| *S. taipeiensis* | Taiwan | Maokong, Taiwan |
| *S. amoena* | Southwestern China | Kunming Institute of Botany, China |
| *S. amabilis* | Japan | Osaka Mt. Ikoma, Japan |
| *S. sessilifolia* | Sichuan, China | Sichuan, China |
| *S. galericulata* | North American | B & T World Seed |
| *S. lateriflora* | North American | B & T World Seed |
| *S. incana* | North American | B & T World Seed |
| *S. alpina* | Europe alpine region | Chiltern Seed |
| *S. baicalensis* | Northeastern Asia | B & T World Seed |
| *S. salviifolia* | Western Asia | Chiltern Seed |
| *S. diffusa* | Western Asia | Chiltern Seed |
| *S. altissima* | Europe | B & T World Seed |
| *S. zhongdianensis* | Europe | B & T World Seed |
| *Holmskioldia sanguinea* | South Asia | Flower market in Pingtung, Taiwan |
| *Tinnea rhodesiana* | South American | B & T World Seed |
