# Supplementary material for: Diversification, Biogeographic Pattern, and Demographic History of Taiwanese Scutellaria Species Inferred from Nuclear and Chloroplast DNA
Source: PLoS One. 2012 Nov 30;7(11):e50844. doi: 10.1371/journal.pone.0050844 (PMC3511331; doi:10.1371/journal.pone.0050844)
Supplement: Table S2 — Best substitution models for the five loci used in the Bayesian analyses. (DOCX) [file pone.0050844.s004.docx]

**Table S2** Best substitution models for the five loci used in the Bayesian analyses.

|  | **Model** | **Parameters** | **BIC** | **AICc** | ***lnL*** | **(+*I*)^a^** | **(+*G*)^b^** | ***R*^c^** |
| --- | --- | --- | --- | --- | --- | --- | --- | --- |
| CAD | HKY+G | 52 | 7063.544 | 6649.751 | -3272.75 | n/a | 0.68 | 1.09 |
| CHS | HKY+I | 50 | 6899.412 | 6509.124 | -3204.42 | 0.66 | n/a | 1.08 |
| matK | HKY | 37 | 2909.655 | 2633.188 | -1279.49 | n/a | n/a | 1.89 |
| ndhF-rpl32 | GTR+G | 60 | 3199.41 | 2748.42 | -1313.94 | n/a | 0.48 | 0.52 |
| rpl32-trnL | HKY+G | 46 | 3522.231 | 3181.719 | -1544.68 | n/a | 0.31 | 0.49 |

^a^Proportion of the invariable sites;

^b^Modeling of discrete gamma distribution;

^c^Transition/transversion bias;

HKY: Hasegawa-Kishino-Yano model

GTR: general time reversible model
