# Supplementary material for: Diversification, Biogeographic Pattern, and Demographic History of Taiwanese Scutellaria Species Inferred from Nuclear and Chloroplast DNA
Source: PLoS One. 2012 Nov 30;7(11):e50844. doi: 10.1371/journal.pone.0050844 (PMC3511331; doi:10.1371/journal.pone.0050844)
Supplement: Table S3 — Tail probabilities of asymmetric values for the among-lineage diversification rate variation in the phylogenetic topologies reconstructed from total samples and Taiwanese samples, respectively, inferred from the species tree (BEAST). (DOCX) [file pone.0050844.s005.docx]

**Table S3** Tail probabilities of asymmetric values for the among-lineage diversification rate variation in the phylogenetic topologies reconstructed from total samples and Taiwanese samples, respectively, inferred from the species tree (BEAST).

|  | *I_C_* | *M_Π_** | *M_Π_* | *M_Σ_** | *M_Σ_* |
| --- | --- | --- | --- | --- | --- |
| Total sample |  |  |  |  |  |
| Min ERM | 136 | -1.516 | 1.84E-10 | 0.268 | 5.879 |
| Max ERM | 6 | 0 | 1 | 1 | 17 |
| Observed | 58 | -0.674 | 4.83E-04 | 0.604 | 12.321 |
| Tail Prob. | 0.030 | 0.044 | 0.035 | 0.033 | 0.026 |
| Taiwan *Scutellaria* |  |  |  |  |  |
| Min ERM | 15 | -0.640 | 0.044 | 0.573 | 3.9 |
| Max ERM | 2.000 | 0 | 1.000 | 1.000 | 6.000 |
| Observed | 9.000 | -0.332 | 0.222 | 0.742 | 4.833 |
| Tail Prob. | 0.333 | 0.378 | 0.333 | 0.222 | 0.333 |

*I_C_* is the Colless’s tree imbalance index; *M_Π_* and *M_Σ_* are the nodal probability product and nodal probability sum of the tree, respectively; *M_Π_** and *M_Σ_** are modified versions of *M_Π_* and *M_Σ_* obtained through differential weighting of the individual equal-rate Markov nodal probabilities according to their species diversity. These indices display the diversification rate variation of the whole tree.
