# Supplementary material for: Diversification, Biogeographic Pattern, and Demographic History of Taiwanese Scutellaria Species Inferred from Nuclear and Chloroplast DNA
Source: PLoS One. 2012 Nov 30;7(11):e50844. doi: 10.1371/journal.pone.0050844 (PMC3511331; doi:10.1371/journal.pone.0050844)
Supplement: Table S4 — Genetic diversity of populations of Scutellaria species in Taiwan estimated using four polymorphic loci. The monomorphic matK in every population is not shown. (DOCX) [file pone.0050844.s006.docx]

**Table S4** Genetic diversity of populations of *Scutellaria* species in Taiwan estimated using four polymorphic loci. The monomorphic *mat*K in every population is not shown.

| Species/Populations | Locus | N | Hap | S | Hd ± std | π ± std | θ ± std | Tajima's D | p | Fu's F | p |
| --- | --- | --- | --- | --- | --- | --- | --- | --- | --- | --- | --- |
| ***S. austrotaiwanensis*** | ***CAD*** | **66‎** | **3** | **2** | **0.671 ± 0.015** | **0.001 ± 4e-5** | **3.7e-4 ± 2.7e-4** | **1.915** | **0.967** | **2.024** | **0.864** |
| aus1 | *CAD* | 19‎ | 1 | 0 | 0 | 0 | 0 | na | na | na | na |
| aus2 | *CAD* | 33‎ | 2 | 1 | 0.477 ± 0.050 | 4.2e-4 ± 4e-5 | 2.2e-4 ± 2.2e-4 | 1.417 | 0.918 | 1.603 | 0.858 |
| aus3 | *CAD* | 14‎ | 1 | 0 | 0 | 0 | 0 | na | na | na | na |
| ***S. indica*** | ***CAD*** | **21‎** | **2** | **6** | **0.514 ± 0.046** | **0.003 ± 2e-4** | **0.001 ± 0.001** | **2.671** | **0.999** | **7.641** | **0.995** |
| ind1 | *CAD* | 9‎ | 1 | 0 | 0 | 0 | 0 | na | na | na | na |
| ind2 | *CAD* | 12‎ | 1 | 0 | 0 | 0 | 0 | na | na | na | na |
| ***S. playfairii*** | ***CAD*** | **83‎** | **3** | **2** | **0.545 ± 0.025** | **0.001 ± 4e-5** | **4e-4 ± 3e-4** | **0.739** | **0.791** | **1.032** | **0.739** |
| pla1 | *CAD* | 5‎ | 1 | 0 | 0 | 0 | 0 | na | na | na | na |
| pla2 | *CAD* | 35‎ | 1 | 0 | 0 | 0 | 0 | na | na | na | na |
| pla3 | *CAD* | 43‎ | 2 | 1 | 0.173 ± 0.072 | 2e-4 ± 6e-5 | 2e-4 ± 2e-4 | -0.353 | 0.241 | 0.063 | 0.451 |
| ***S. taiwanensis*** | ***CAD*** | **31‎** | **1** | **0** | **0** | **0** | **0** | **na** | **na** | **na** | **na** |
| ***S. tashiroi*** | ***CAD*** | **68‎** | **3** | **2** | **0.648 ± 0.025** | **0.001 ± 3e-5** | **4e-4 ± 3e-4** | **2.111** | **0.979** | **2.203** | **0.890** |
| tas1 | *CAD* | 15‎ | 1 | 0 | 0 | 0 | 0 | na | na | na | na |
| tas2 | *CAD* | 16‎ | 1 | 0 | 0 | 0 | 0 | na | na | na | na |
| tas3 | *CAD* | 15‎ | 1 | 0 | 0 | 0 | 0 | na | na | na | na |
| tas4 | *CAD* | 22‎ | 1 | 0 | 0 | 0 | 0 | na | na | na | na |
| ***S. austrotaiwanensis*** | ***CHS*** | **132‎** | **2** | **1** | **0.413 ± 0.034** | **0.001 ± 4e-5** | **2e-4 ± 2e-4** | **1.363** | **0.900** | **2.011** | **0.864** |
| aus1 | *CHS* | 38‎ | 1 | 0 | 0 | 0 | 0 | na | na | na | na |
| aus2 | *CHS* | 66‎ | 1 | 0 | 0 | 0 | 0 | na | na | na | na |
| aus3 | *CHS* | 28‎ | 1 | 0 | 0 | 0 | 0 | na | na | na | na |
| ***S. indica*** | ***CHS*** | **42‎** | **2** | **4** | **0.502 ± 0.027** | **0.003 ± 1e-4** | **0.001 ± 0.001** | **2.735** | **0.997** | **6.824** | **0.989** |
| ind1 | *CHS* | 18‎ | 1 | 0 | 0 | 0 | 0 | na | na | na | na |
| ind2 | *CHS* | 24‎ | 1 | 0 | 0 | 0 | 0 | na | na | na | na |
| ***S. playfairii*** | ***CHS*** | **166‎** | **2** | **1** | **0.491 ± 0.013** | **0.001 ± 2e-5** | **2e-4 ± 2e-4** | **1.868** | **0.961** | **2.554** | **0.908** |
| pla1 | *CHS* | 10‎ | 1 | 0 | 0 | 0 | 0 | na | na | na | na |
| pla2 | *CHS* | 70‎ | 1 | 0 | 0 | 0 | 0 | na | na | na | na |
| pla3 | *CHS* | 86‎ | 1 | 0 | 0 | 0 | 0 | na | na | na | na |
| ***S. taiwanensis*** | ***CHS*** | **62‎** | **1** | **0** | **0** | **0** | **0** | **na** | **na** | **na** | **na** |
| ***S. tashiroi*** | ***CHS*** | **136‎** | **6** | **4** | **0.652 ± 0.033** | **0.002 ± 1e-4** | **0.001 ± 0.001** | **2.078** | **0.977** | **1.362** | **0.764** |
| tas1 | *CHS* | 30‎ | 1 | 0 | 0 | 0 | 0 | na | na | na | na |
| tas2 | *CHS* | 32‎ | 1 | 0 | 0 | 0 | 0 | na | na | na | na |
| tas3 | *CHS* | 30‎ | 2 | 1 | 0.460 ± 0.061 | 0.001 ± 8e-5 | 3e-4 ± 3e-4 | 1.280 | 0.897 | 1.475 | 0.836 |
| tas4 | *CHS* | 44‎ | 4 | 3 | 0.529 ± 0.065 | 0.001 ± 1e-4 | 0.001 ± 0.001 | -0.328 | 0.384 | -0.563 | 0.366 |
| ***S. austrotaiwanensis*** | ***ndh*F-*rpl*32** | **66‎** | **1** | **0** | **0** | **0** | **0** | **na** | **na** | **na** | **na** |
| aus1 | *ndh*F-*rpl*32 | 19‎ | 1 | 0 | 0 | 0 | 0 | na | na | na | na |
| aus2 | *ndh*F-*rpl*32 | 33‎ | 1 | 0 | 0 | 0 | 0 | na | na | na | na |
| aus3 | *ndh*F-*rpl*32 | 14‎ | 1 | 0 | 0 | 0 | 0 | na | na | na | na |
| ***S. indica*** | ***ndh*F-*rpl*32** | **21‎** | **2** | **3** | **0.514 ± 0.046** | **0.003 ± 2e-4** | **0.001 ± 0.001** | **2.234** | **0.991** | **4.389** | **0.975** |
| ind1 | *ndh*F-*rpl*32 | 9‎ | 1 | 0 | 0 | 0 | 0 | na | na | na | na |
| ind2 | *ndh*F-*rpl*32 | 12‎ | 1 | 0 | 0 | 0 | 0 | na | na | na | na |
| ***S. playfairii*** | ***ndh*F-*rpl*32** | **83‎** | **3** | **3** | **0.360 ± 0.058** | **0.001 ± 2e-4** | **0.001 ± 0.001** | **0.184** | **0.636** | **1.328** | **0.791** |
| pla1 | *ndh*F-*rpl*32 | 5‎ | 1 | 0 | 0 | 0 | 0 | na | na | na | na |
| pla2 | *ndh*F-*rpl*32 | 35‎ | 2 | 1 | 0.208 ± 0.083 | 4e-4 ± 2e-4 | 4e-4 ± 4e-4 | -0.211 | 0.285 | 0.251 | 0.503 |
| pla3 | *ndh*F-*rpl*32 | 43‎ | 2 | 2 | 0.450 ± 0.520 | 0.002 ± 2e-4 | 0.001 ± 0.001 | 1.752 | 0.958 | 3.387 | 0.952 |
| ***S. taiwanensis*** | ***ndh*F-*rpl*32** | **31‎** | **2** | **1** | **0.452 ± 0.063** | **0.001 ± 1e-4** | **4e-4 ± 4e-4** | **1.240** | **0.884** | **1.459** | **0.824** |
| ***S. tashiroi*** | ***ndh*F-*rpl*32** | **68‎** | **5** | **5** | **0.717 ± 0.038** | **0.003 ± 3e-4** | **0.002 ± 0.001** | **1.763** | **0.963** | **2.215** | **0.852** |
| tas1 | *ndh*F-*rpl*32 | 15‎ | 1 | 0 | 0 | 0 | 0 | na | na | na | na |
| tas2 | *ndh*F-*rpl*32 | 16‎ | 1 | 0 | 0 | 0 | 0 | na | na | na | na |
| tas3 | *ndh*F-*rpl*32 | 15‎ | 1 | 0 | 0 | 0 | 0 | na | na | na | na |
| tas4 | *ndh*F-*rpl*32 | 22‎ | 3 | 2 | 0.680 ± 0.043 | 0.002 ± 2e-4 | 0.001 ± 0.001 | 1.345 | 0.908 | 1.004 | 0.758 |
| ***S. austrotaiwanensis*** | ***rpl*32-*trn*L** | **66‎** | **1** | **0** | **0** | **0** | **0** | **na** | **na** | **na** | **na** |
| aus1 | *rpl*32-*trn*L | 19‎ | 1 | 0 | 0 | 0 | 0 | na | na | na | na |
| aus2 | *rpl*32-*trn*L | 33‎ | 1 | 0 | 0 | 0 | 0 | na | na | na | na |
| aus3 | *rpl*32-*trn*L | 14‎ | 1 | 0 | 0 | 0 | 0 | na | na | na | na |
| ***S. indica*** | ***rpl*32-*trn*L** | **21‎** | **2** | **6** | **0.514 ± 0.046** | **0.005 ± 5e-4** | **0.003 ± 0.001** | **2.671** | **0.999** | **7.641** | **0.995** |
| ind1 | *rpl*32-*trn*L | 9‎ | 1 | 0 | 0 | 0 | 0 | na | na | na | na |
| ind2 | *rpl*32-*trn*L | 12‎ | 1 | 0 | 0 | 0 | 0 | na | na | na | na |
| ***S. playfairii*** | ***rpl*32-*trn*L** | **83‎** | **3** | **6** | **0.626 ± 0.023** | **0.005 ± 1e-4** | **0.002 ± 0.001** | **3.018** | **0.999** | **8.057** | **0.986** |
| pla1 | *rpl*32-*trn*L | 5‎ | 1 | 0 | 0 | 0 | 0 | na | na | na | na |
| pla2 | *rpl*32-*trn*L | 35‎ | 2 | 6 | 0.363 ± 0.078 | 0.004 ± 0.001 | 0.003 ± 0.001 | 1.361 | 0.920 | 6.916 | 0.992 |
| pla3 | *rpl*32-*trn*L | 43‎ | 2 | 5 | 0.365 ± 0.070 | 0.003 ± 0.001 | 0.002 ± 0.001 | 1.455 | 0.928 | 6.349 | 0.988 |
| ***S. taiwanensis*** | ***rpl*32-*trn*L** | **31‎** | **1** | **0** | **0** | **0** | **0** | **na** | **na** | **na** | **na** |
| ***S. tashiroi*** | ***rpl*32-*trn*L** | **68‎** | **2** | **3** | **0.504 ± 0.015** | **0.003 ± 8e-5** | **0.001 ± 0.001** | **2.728** | **0.996** | **6.085** | **0.982** |
| tas1 | *rpl*32-*trn*L | 15‎ | 1 | 0 | 0 | 0 | 0 | na | na | na | na |
| tas2 | *rpl*32-*trn*L | 16‎ | 1 | 0 | 0 | 0 | 0 | na | na | na | na |
| tas3 | *rpl*32-*trn*L | 15‎ | 1 | 0 | 0 | 0 | 0 | na | na | na | na |
| tas4 | *rpl*32-*trn*L | 22‎ | 1 | 0 | 0 | 0 | 0 | na | na | na | na |
